# Supplementary material for: Cu–Al mixed oxide-catalysed multi-component synthesis of gluco- and allofuranose-linked 1,2,3-triazole derivatives
Source: R Soc Open Sci. 2020 Jul 22;7(7):200290. doi: 10.1098/rsos.200290 (PMC7428250; doi:10.1098/rsos.200290)
Supplement: Copies of 1H and 13C of synthetized compounds [file rsos200290supp1.pdf]

Supporting information

Cu-Al mixed oxide-catalyzed multicomponent synthesis of gluco- and allofuranose-linked 1,2,3-triazole derivatives

Ricardo Corona-Sánchez,[a] Alma Sánchez-Eleuterio,[b] Claudia Negrón-Lomas,[c] Yarisel Ruiz Almazan,[c] Leticia Lomas-Romero,*[,a] Guillermo E. Negrón-Silva,*[,b] Álvaro C. Rodríguez- Sánchez[c]

[a] *Departamento de Química, Universidad Autónoma Metropolitana-Iztapalapa, Av. San Rafael Atlixco 186, Leyes de Reforma 1ra Secc., 09340 Ciudad de México, México*

[b] *Departamento de Ciencias Básicas, Universidad Autónoma Metropolitana-Azcapotzalco, Av. San Pablo No. 180, Ciudad de México, C.P. 02200, México*

[c] *Departamento de Biotecnología, Instituto Tecnológico de Estudios Superiores de Monterrey, Calle del Puente 222, Ciudad de México, C.P. 14380, México*

Contents	Page
^{1}H and ^{13}C spectra for compound **7a**	**S-2**
^{1}H and ^{13}C spectra for compound **7b**	**S-3**
^{1}H and ^{13}C spectra for compound **7c**	**S-4**
^{1}H and ^{13}C spectra for compound **7d**	**S-5**
^{1}H and ^{13}C spectra for compound **7e**	**S-6**
^{1}H and ^{13}C spectra for compound **8a**	**S-7**
^{1}H and ^{13}C spectra for compound **8b**	**S-8**
^{1}H and ^{13}C spectra for compound **8c**	**S-9**
^{1}H and ^{13}C spectra for compound **8d**	**S-10**
^{1}H and ^{13}C spectra for compound **8e**	**S-11**
^{1}H and ^{13}C spectra for compound **9a**	**S-12**
^{1}H and ^{13}C spectra for compound **9b**	**S-13**
^{1}H and ^{13}C spectra for compound **9c**	**S-14**
^{1}H and ^{13}C spectra for compound **9d**	**S-15**
^{1}H and ^{13}C spectra for compound **9e**	**S-16**
^{1}H and ^{13}C spectra for compound **10a**	**S-17**
^{1}H and ^{13}C spectra for compound **10b**	**S-18**
^{1}H and ^{13}C spectra for compound **10c**	**S-19**
^{1}H and ^{13}C spectra for compound **10d**	**S-20**
^{1}H and ^{13}C spectra for compound **10e**	**S-21**

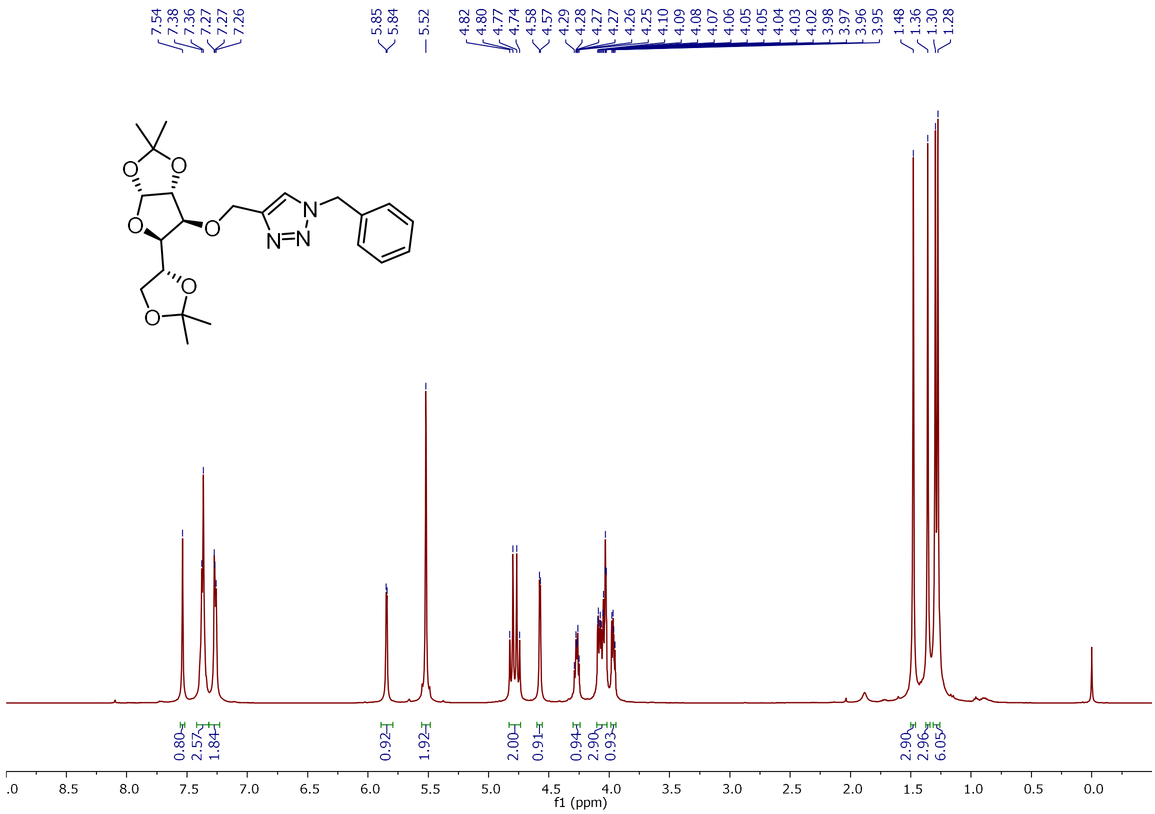

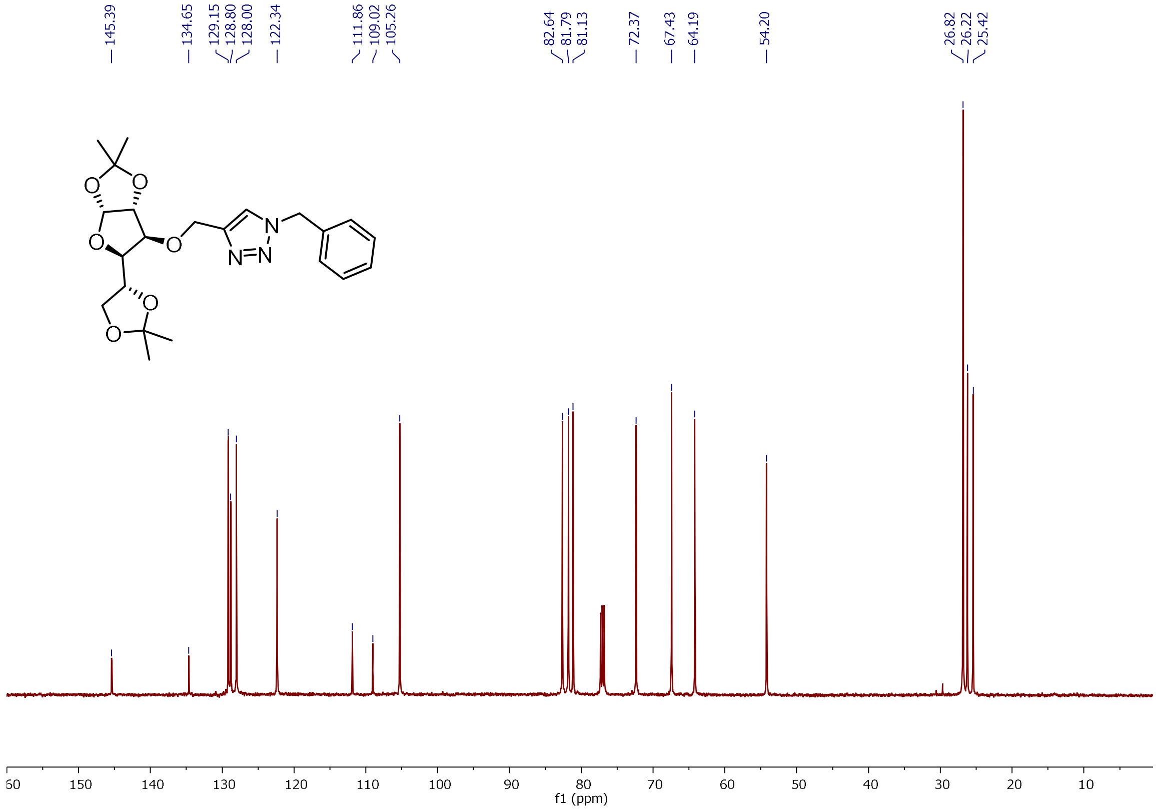

^{1}H and ^{13}C ($CDCl_3$) spectra for compounds **7a**

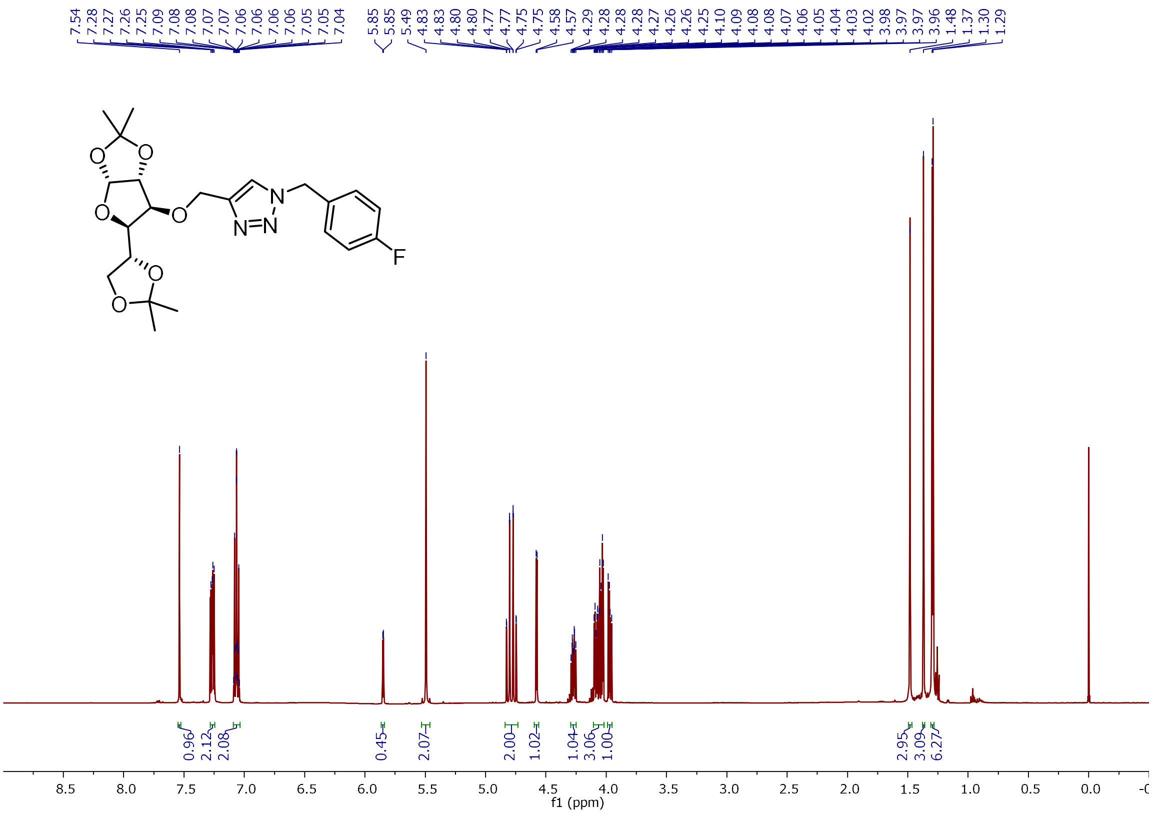

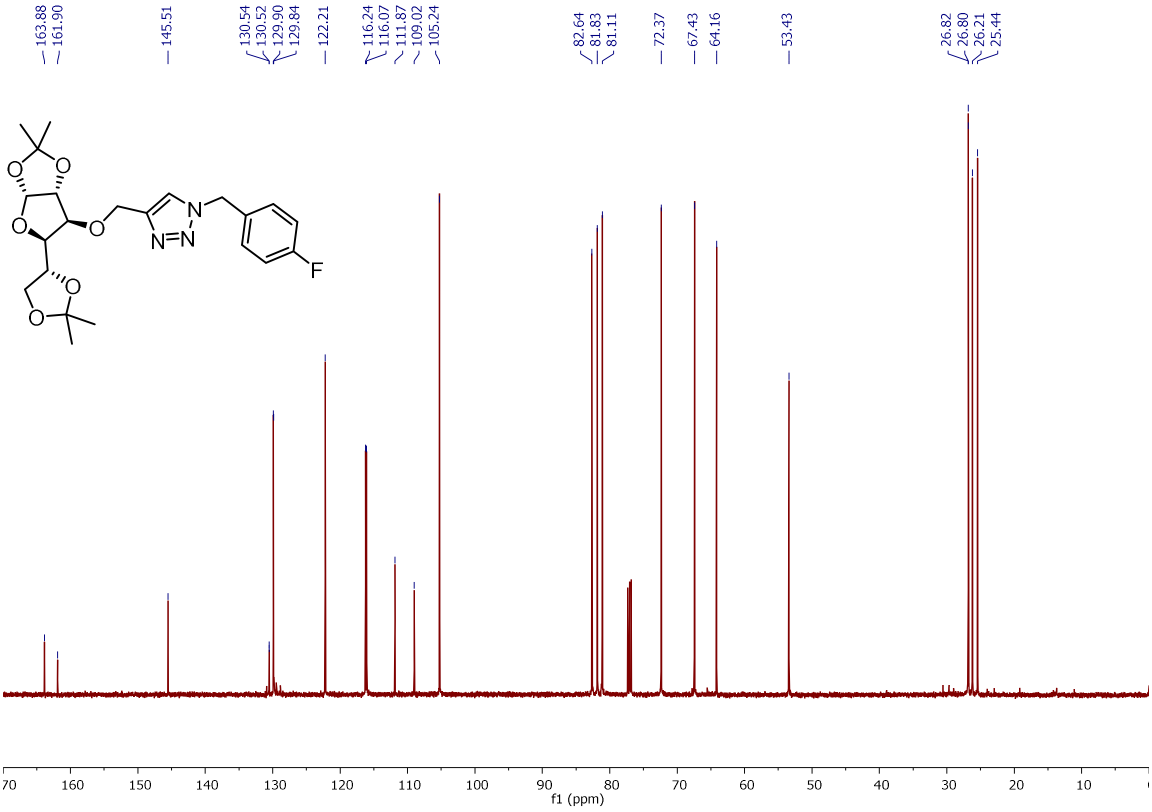

^{1}H and ^{13}C ($CDCl_3$) spectra for compounds **7b**

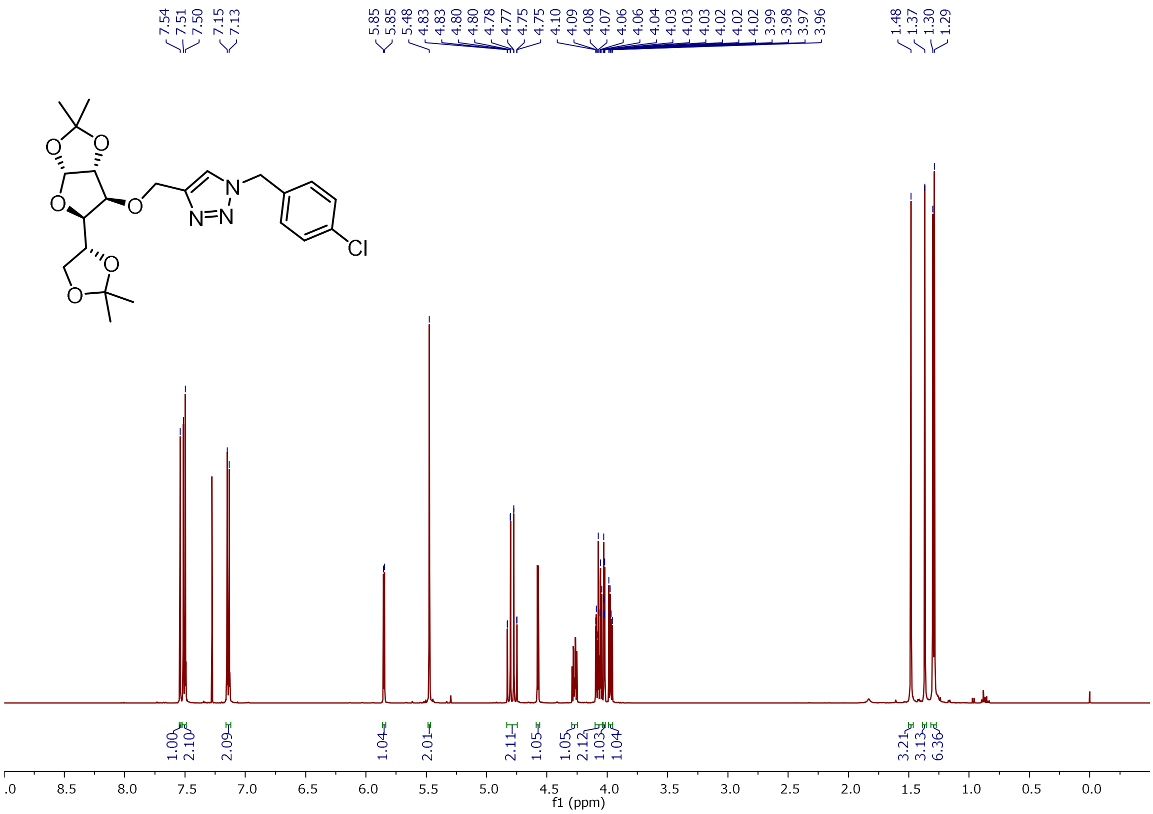

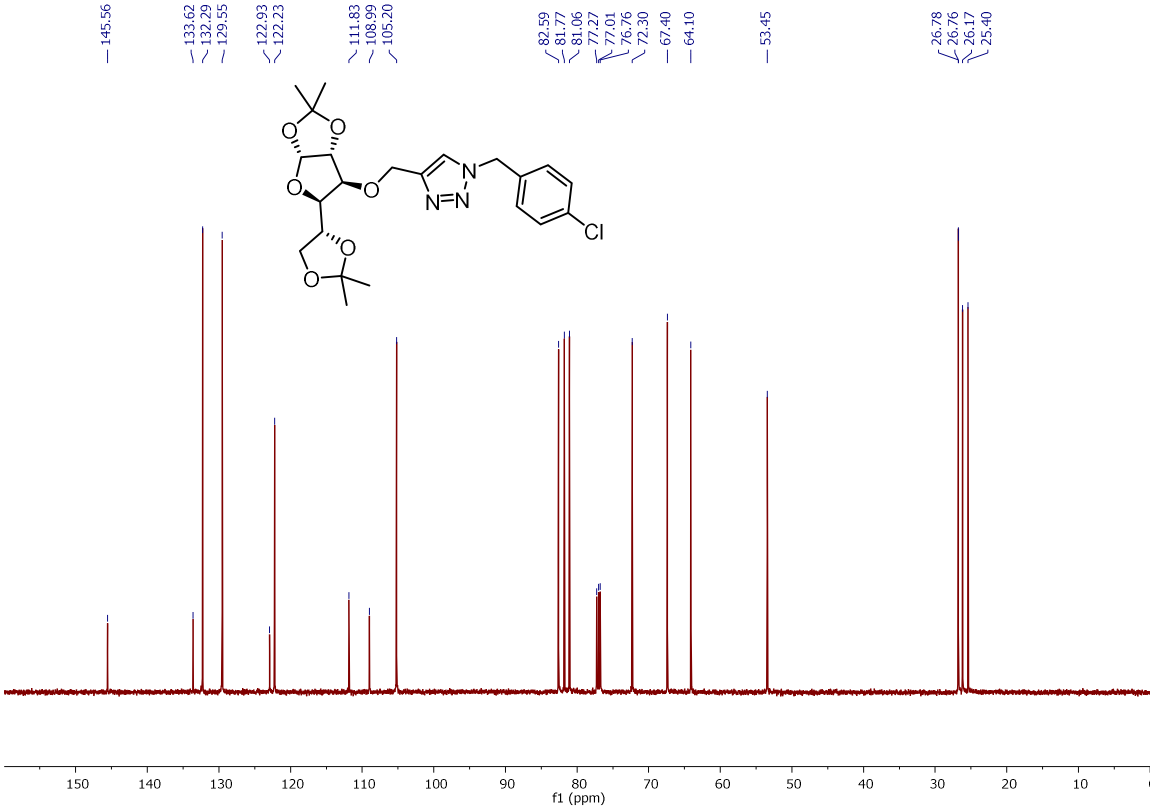

^{1}H and ^{13}C ($CDCl_3$) spectra for compounds **7c**

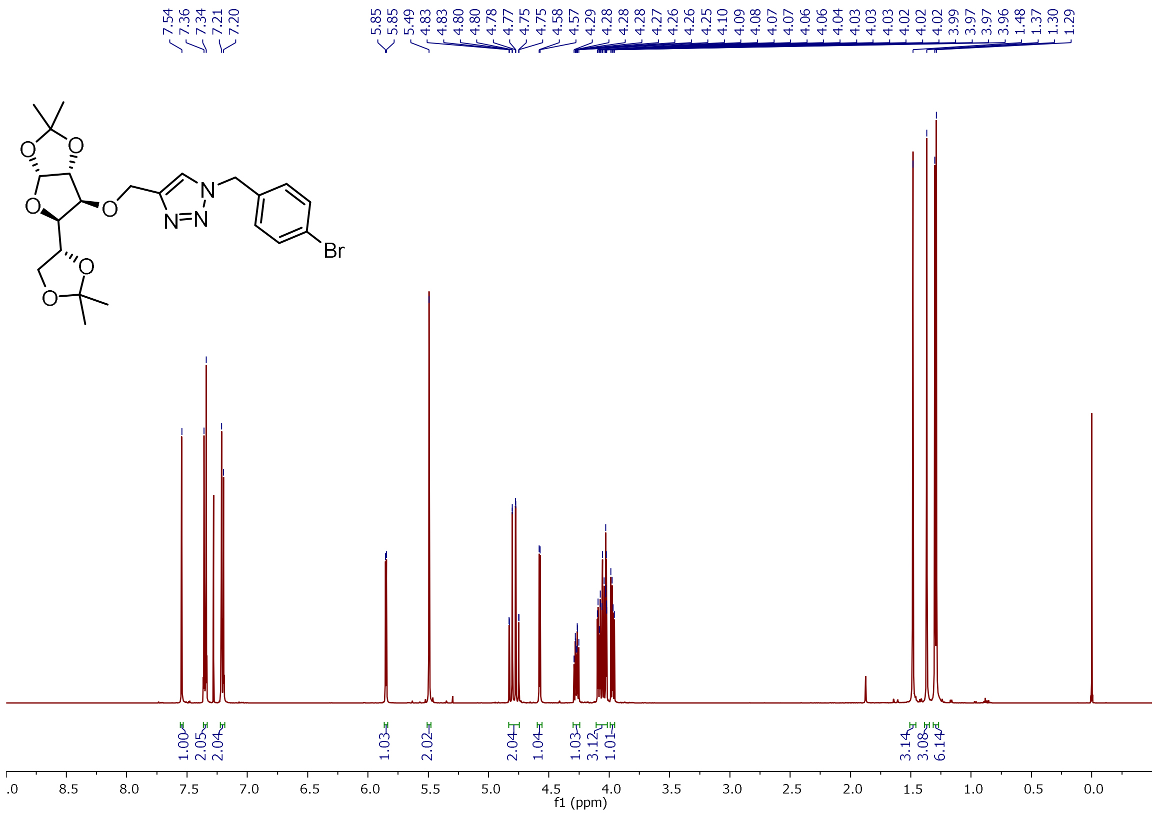

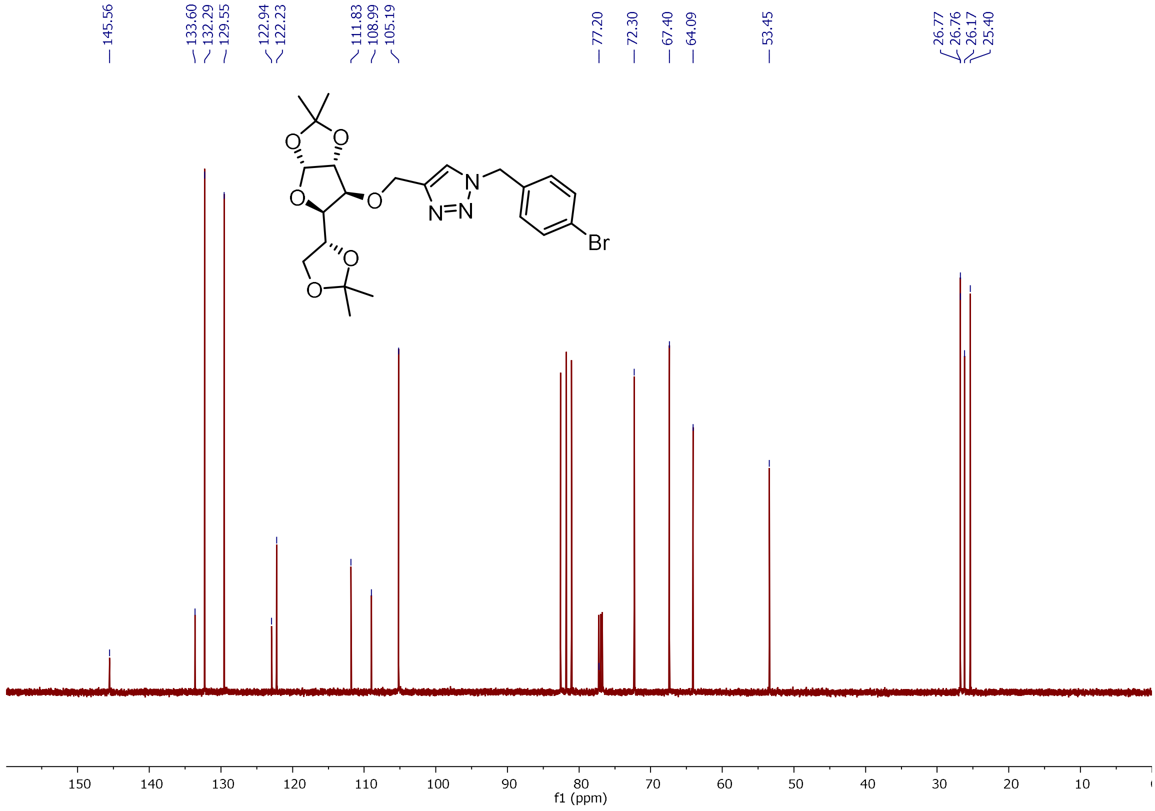

^1H and ^{13}C ($CDCl_3$) spectra for compounds **7d**

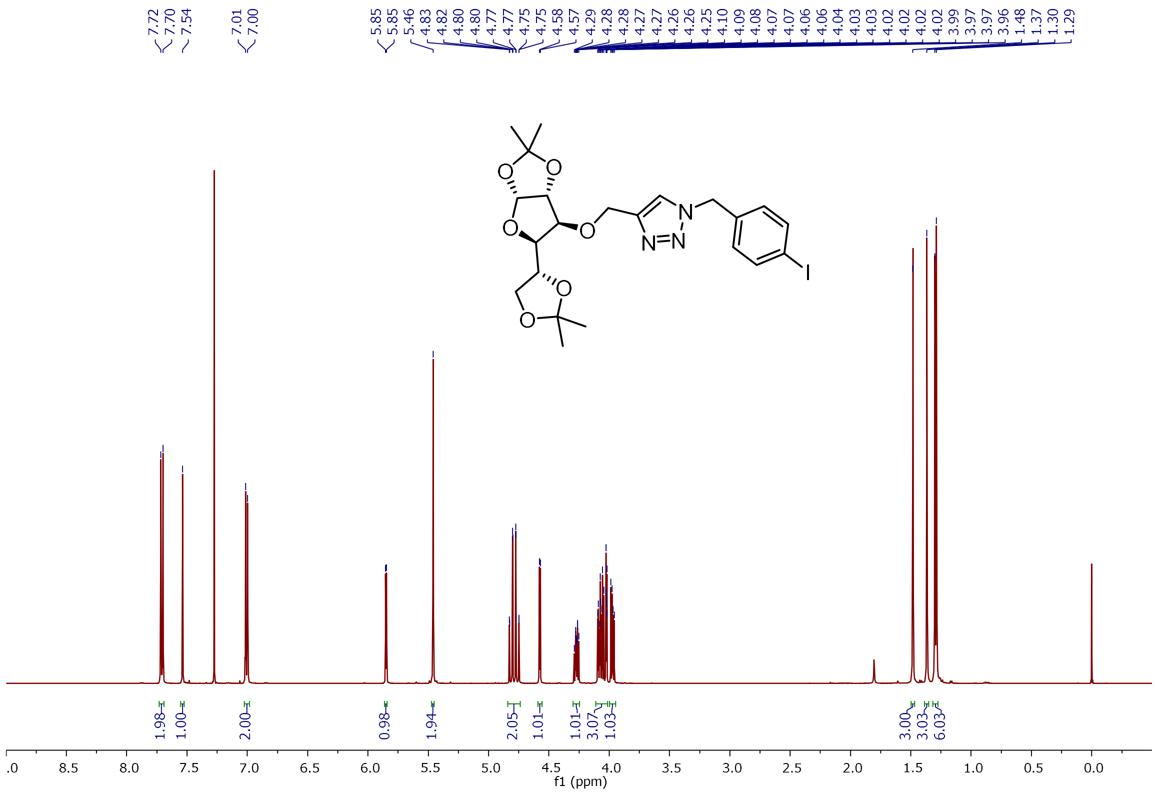

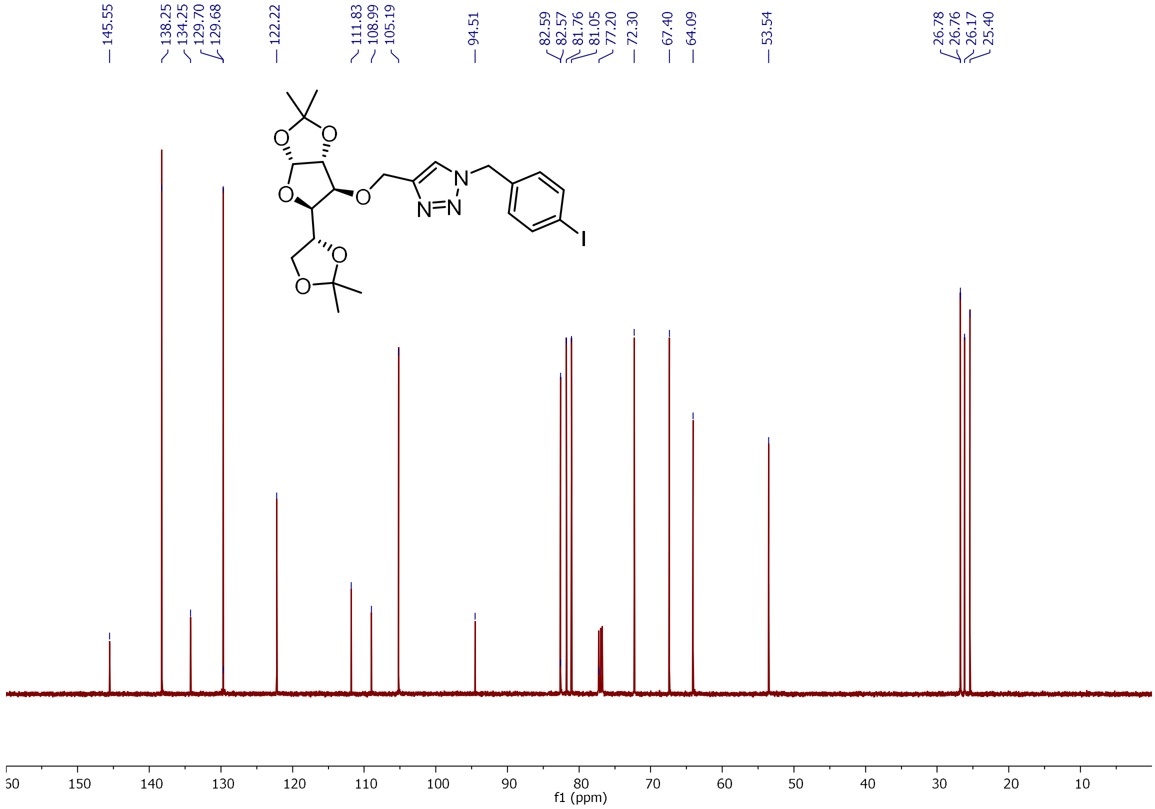

^{1}H and ^{13}C ($CDCl_3$) spectra for compounds **7e**

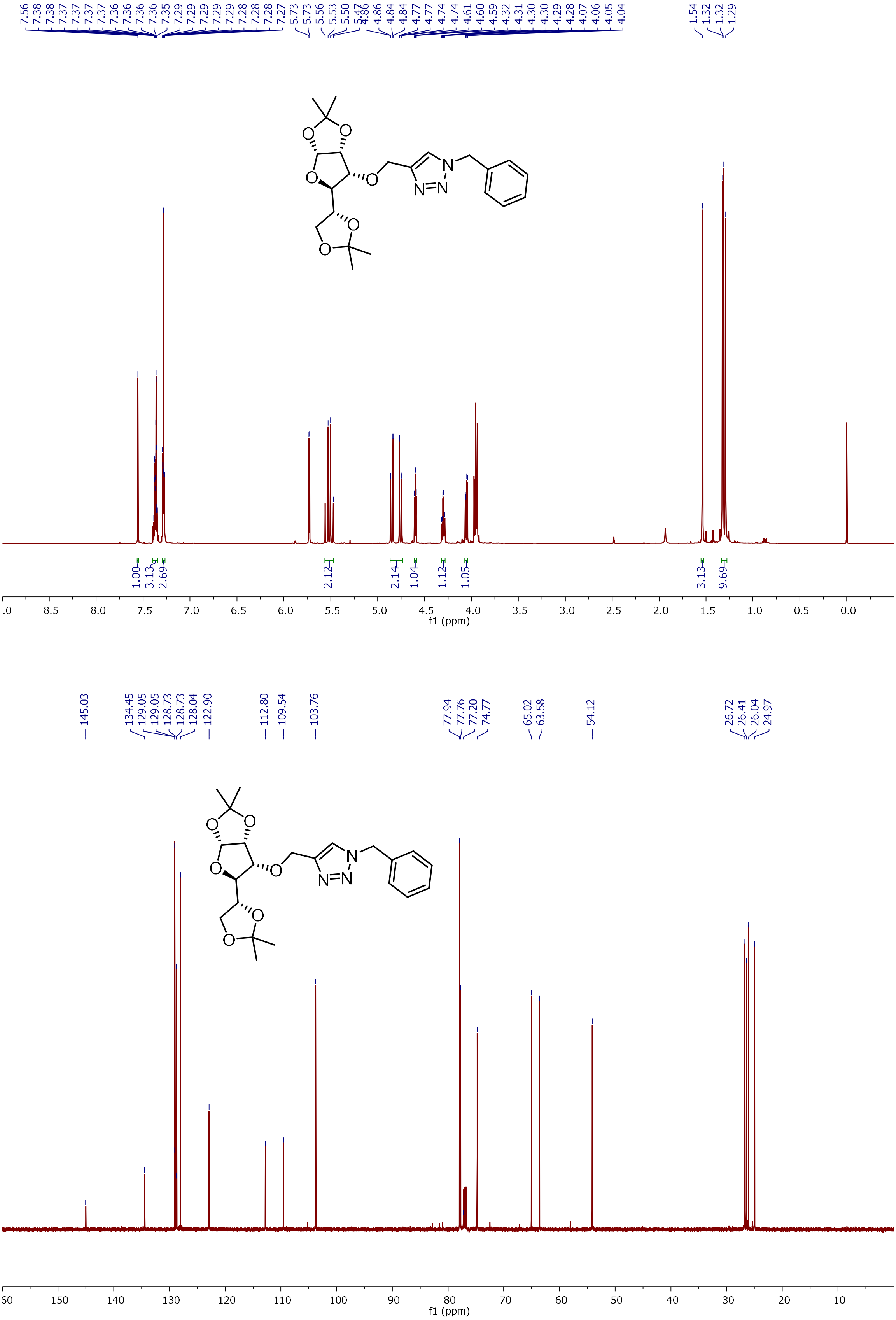

^{1}H and ^{13}C ($CDCl_3$) spectra for compounds **8a**

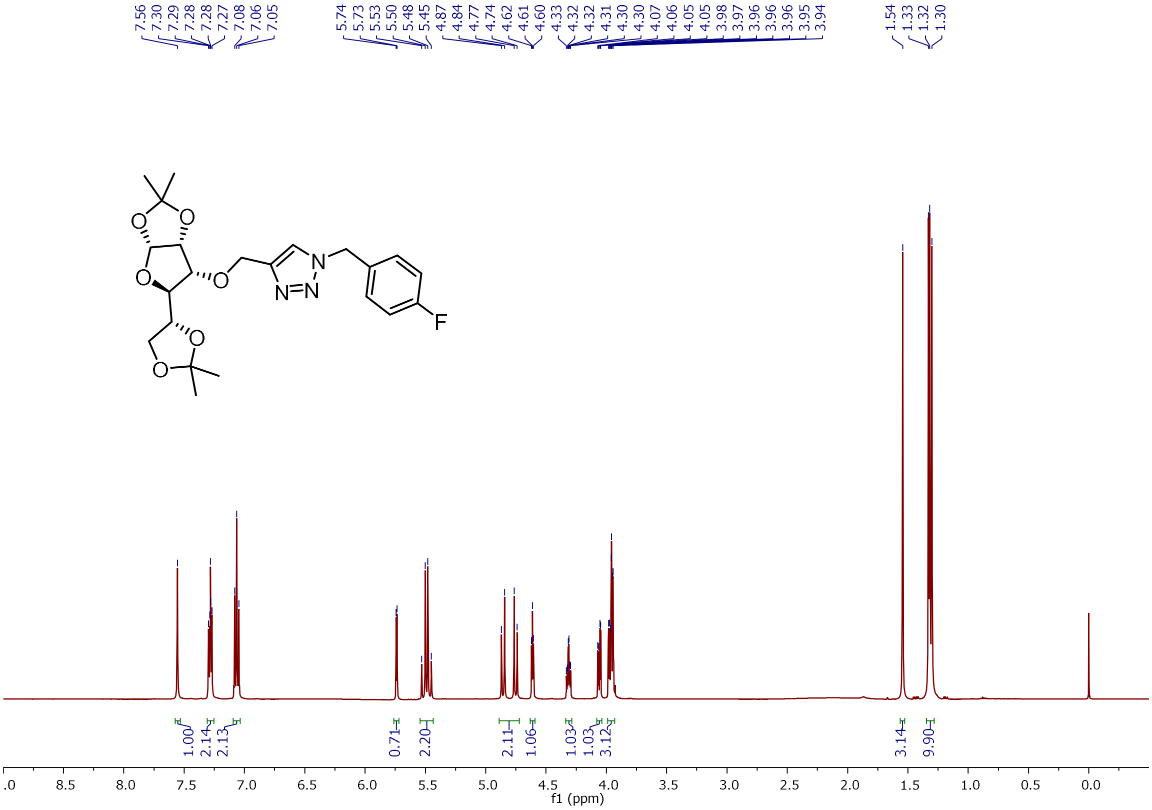

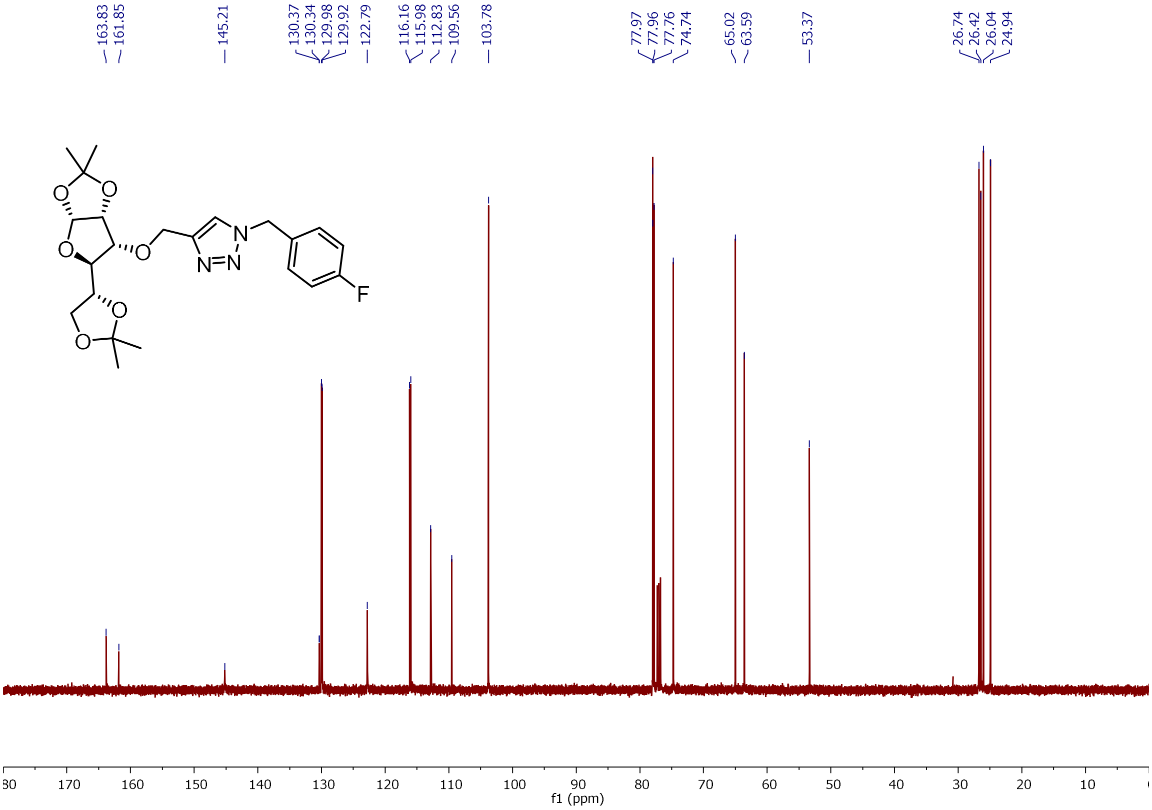

^{1}H and ^{13}C ($CDCl_3$) spectra for compounds **8b**

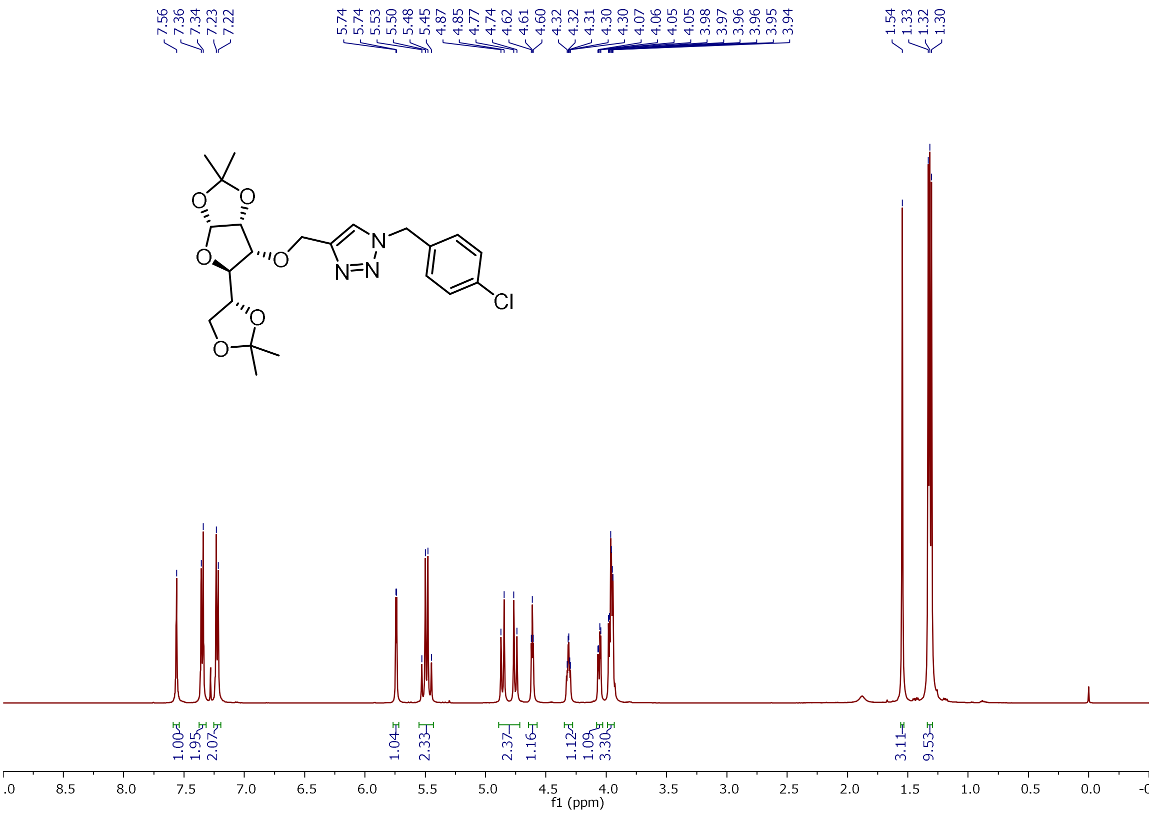

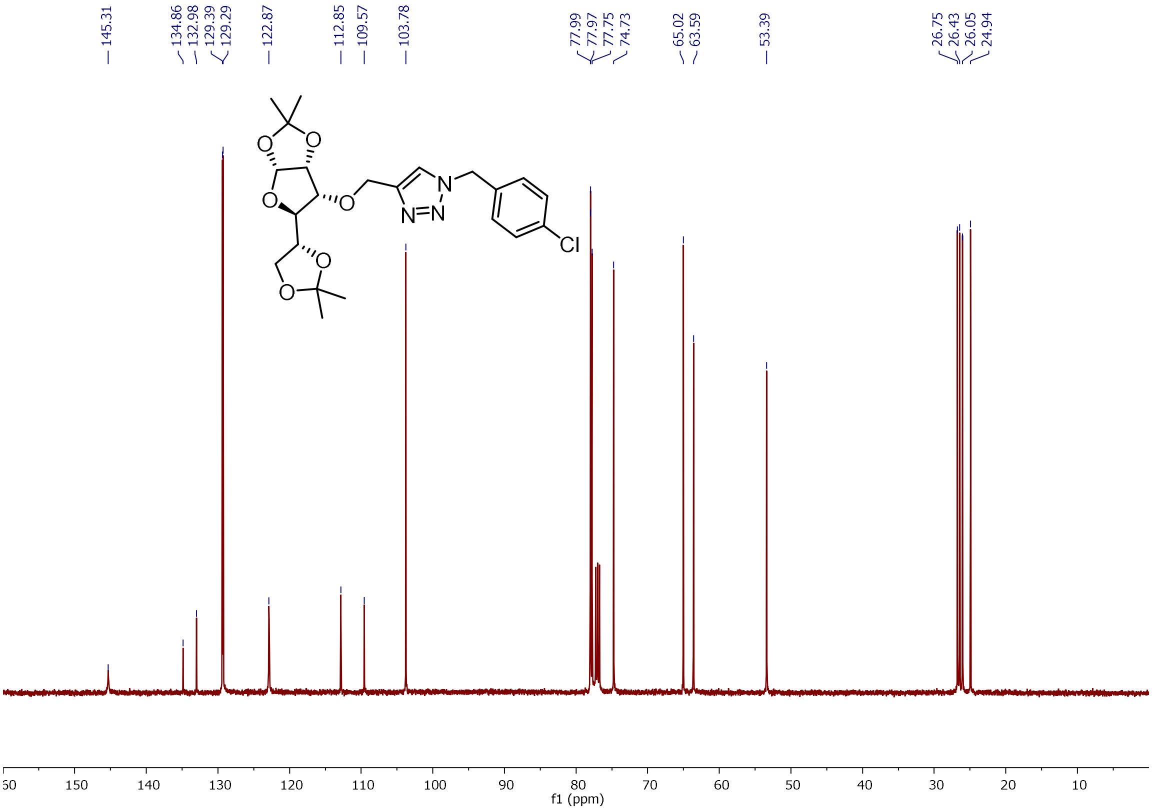

^{1}H and ^{13}C ($CDCl_3$) spectra for compounds **8c**

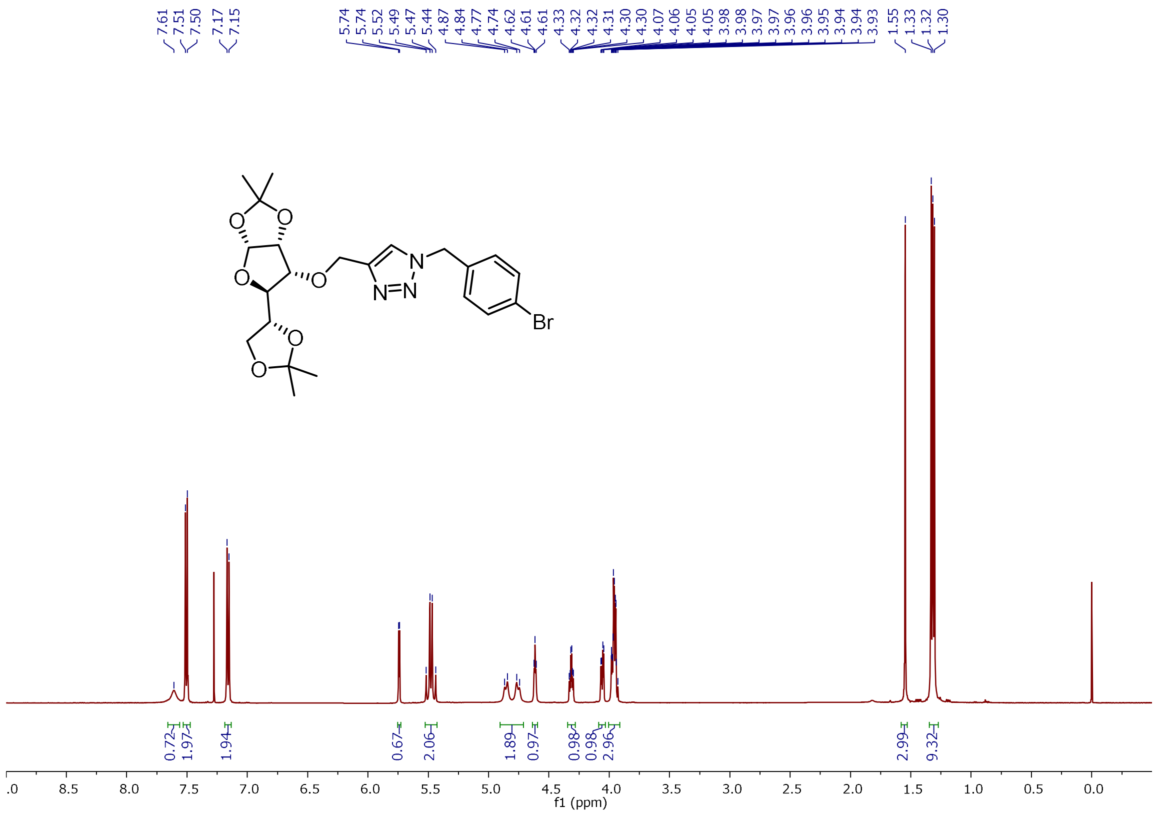

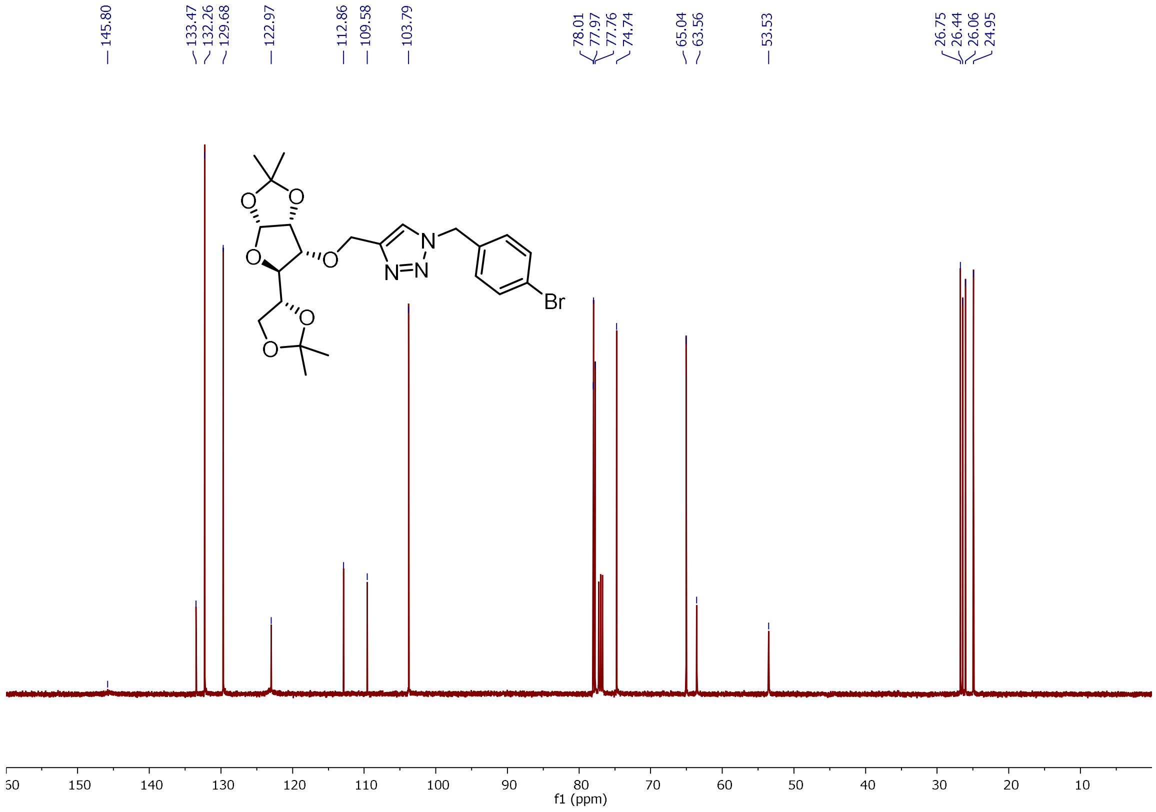

^{1}H and ^{13}C ($CDCl_3$) spectra for compounds **8d**

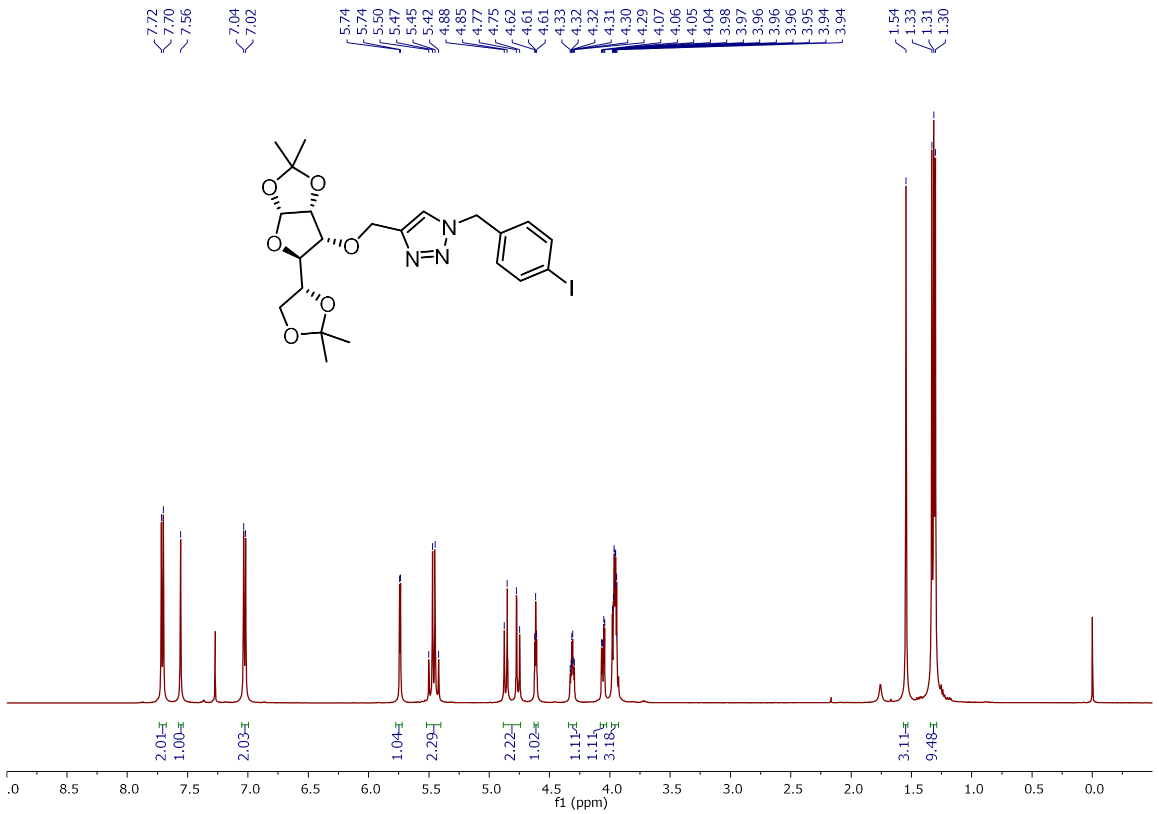

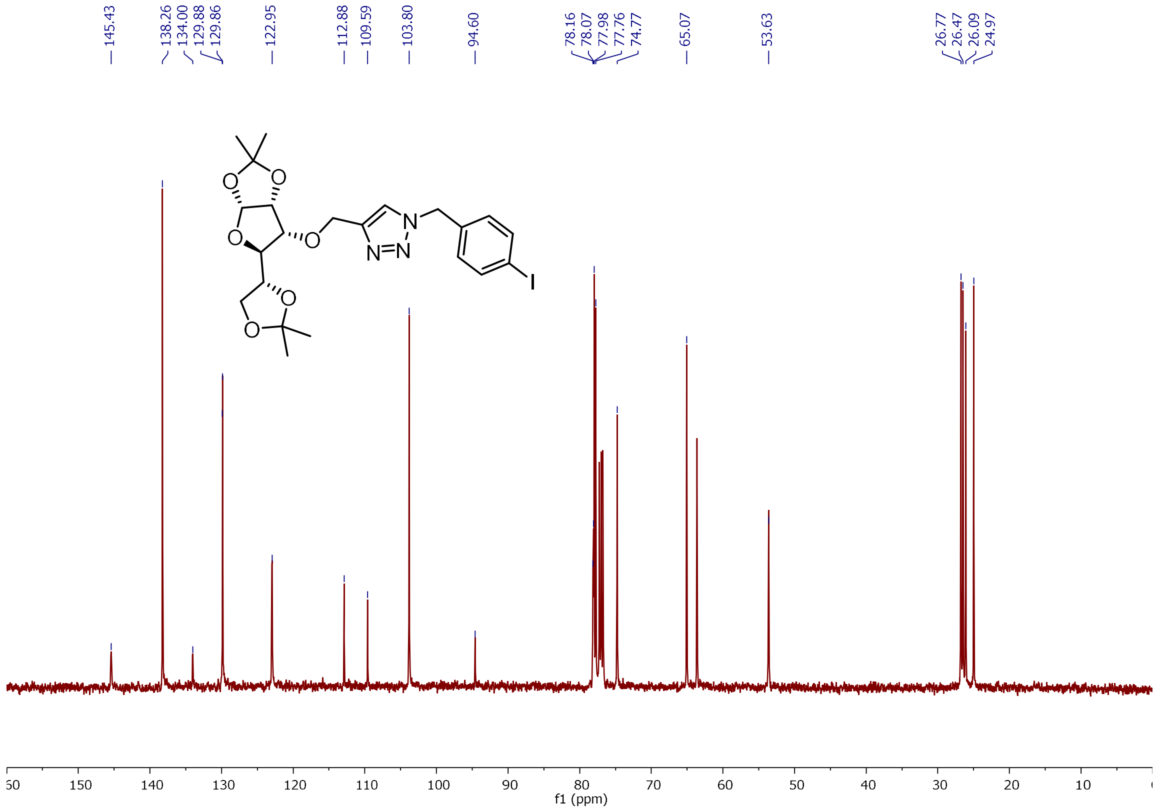

^{1}H and ^{13}C ($CDCl_3$) spectra for compounds **8e**

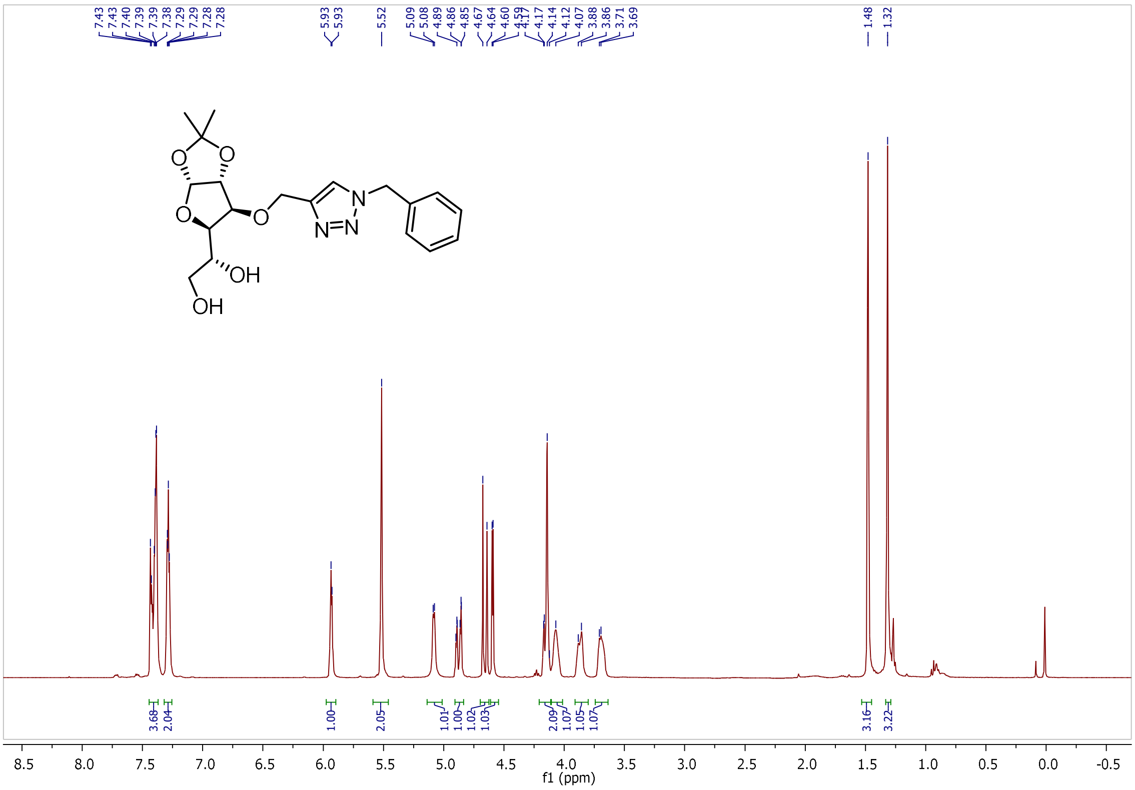

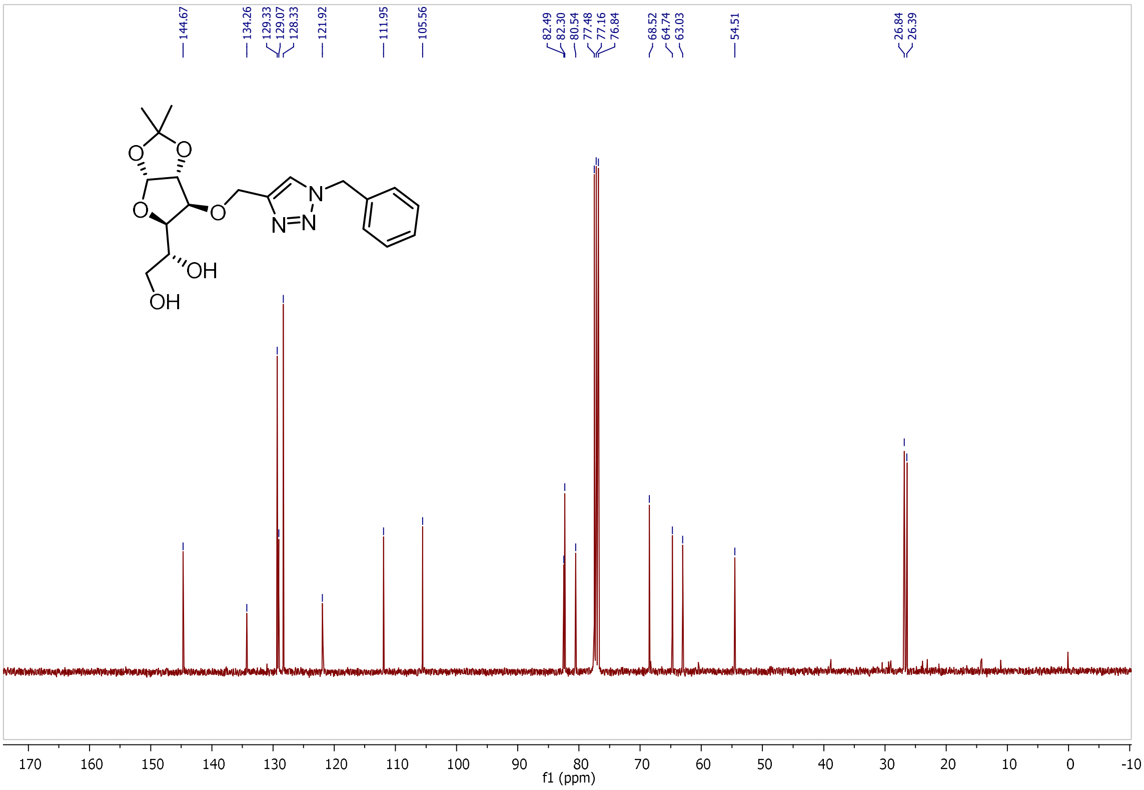

^{1}H and ^{13}C ($CDCl_3$) spectra for compounds **9a**

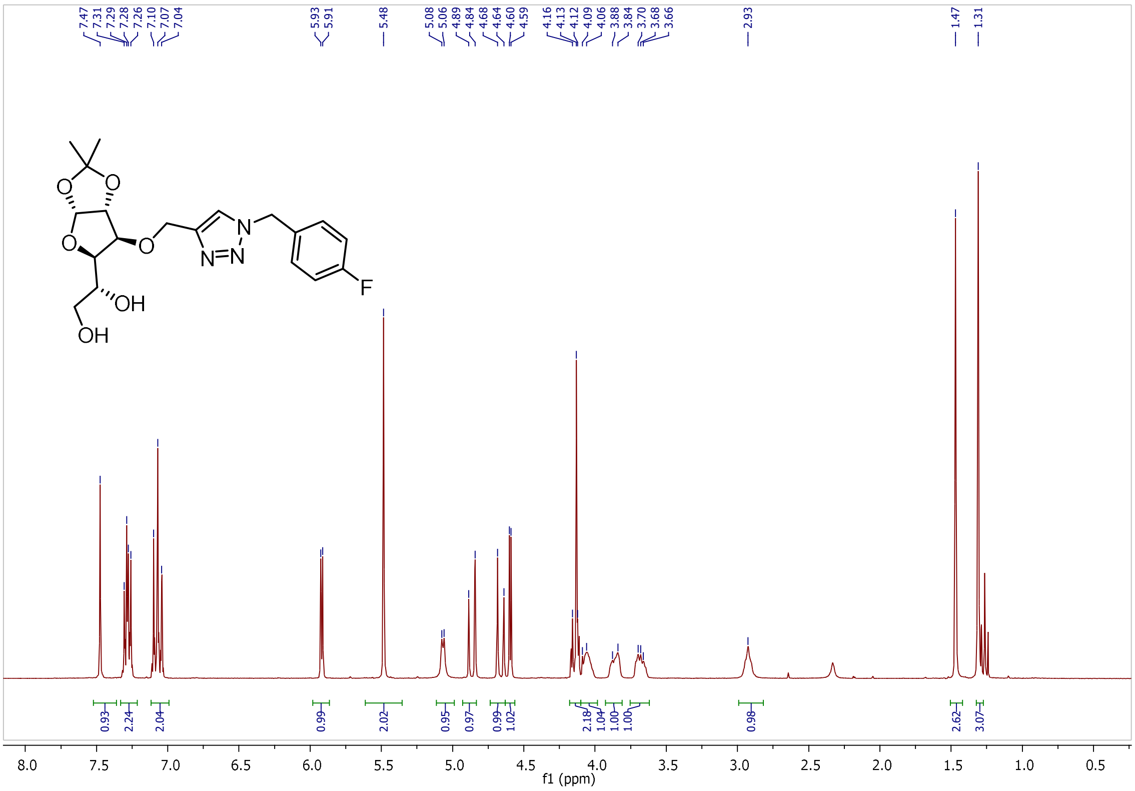

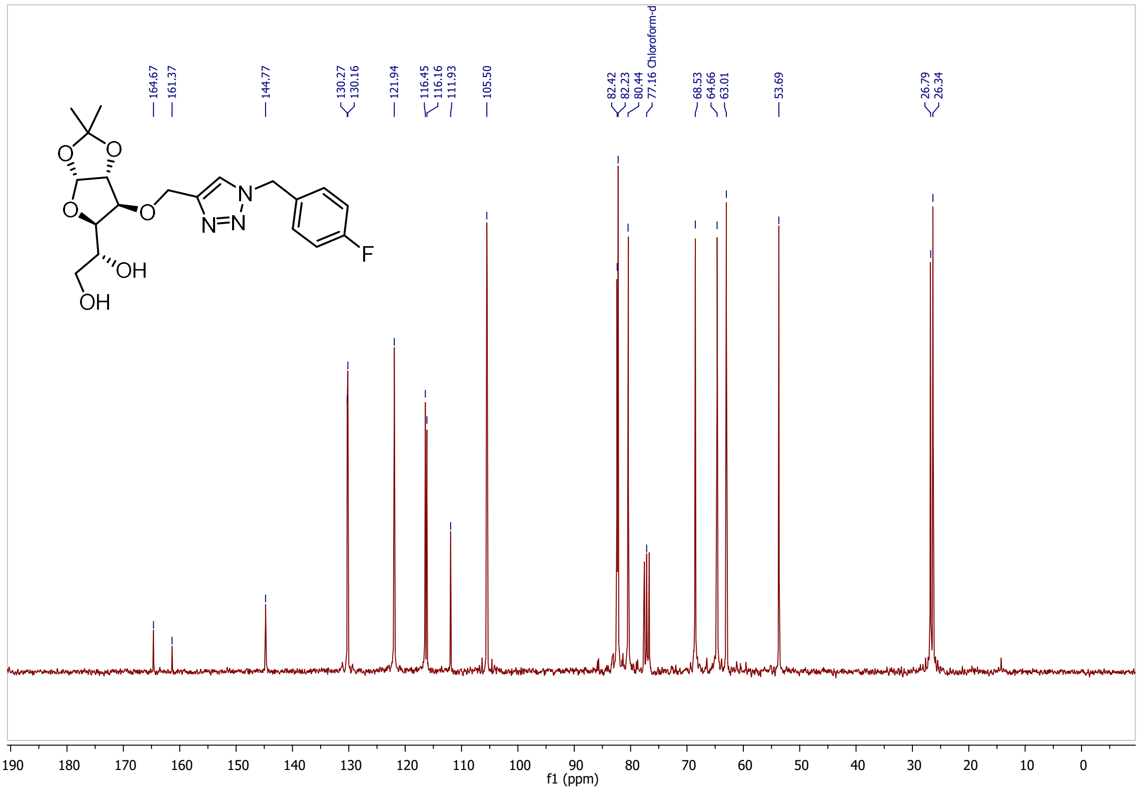

^{1}H and ^{13}C ($CDCl_3$) spectra for compounds **9b**

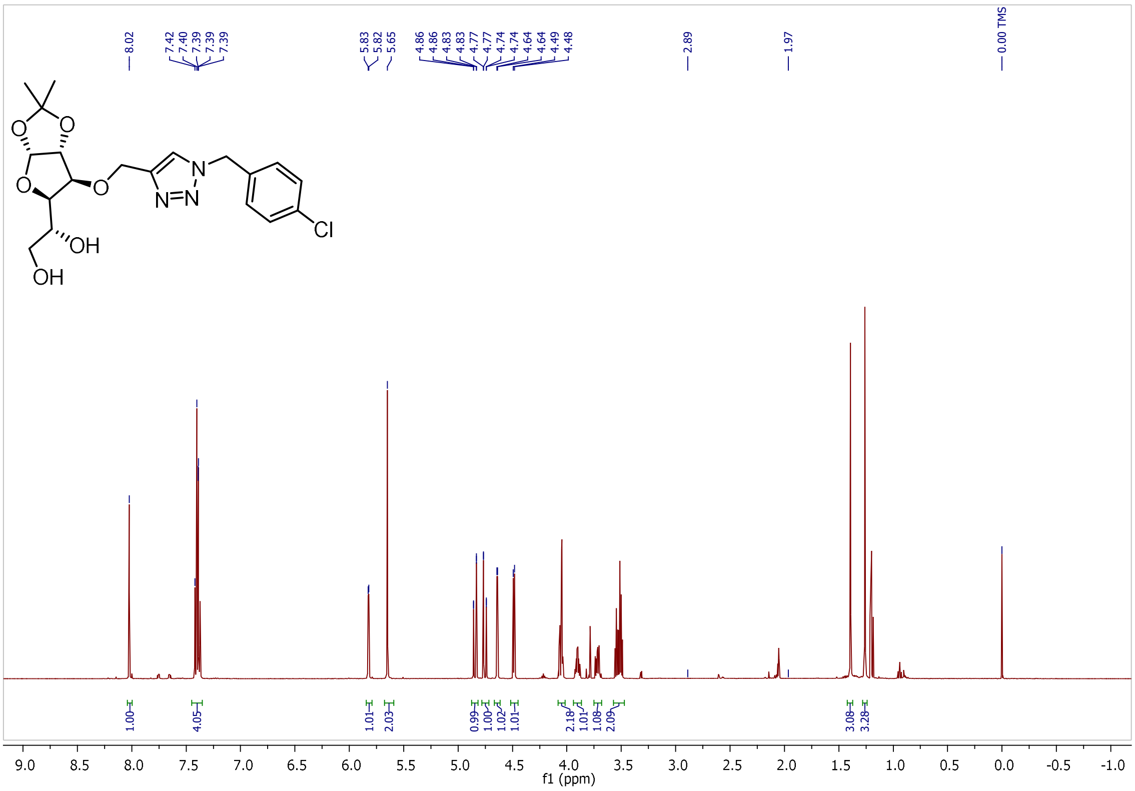

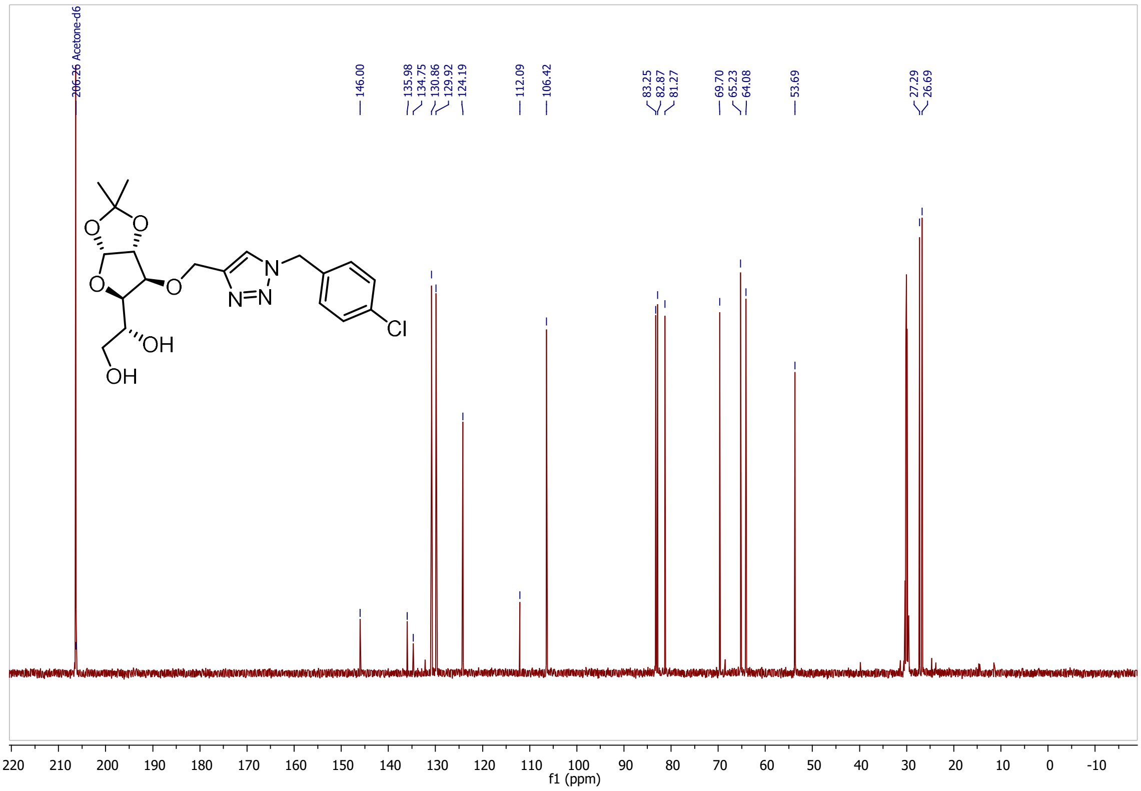

^{1}H and ^{13}C (Acetone-*d$_6$*) spectra for compounds **9c**

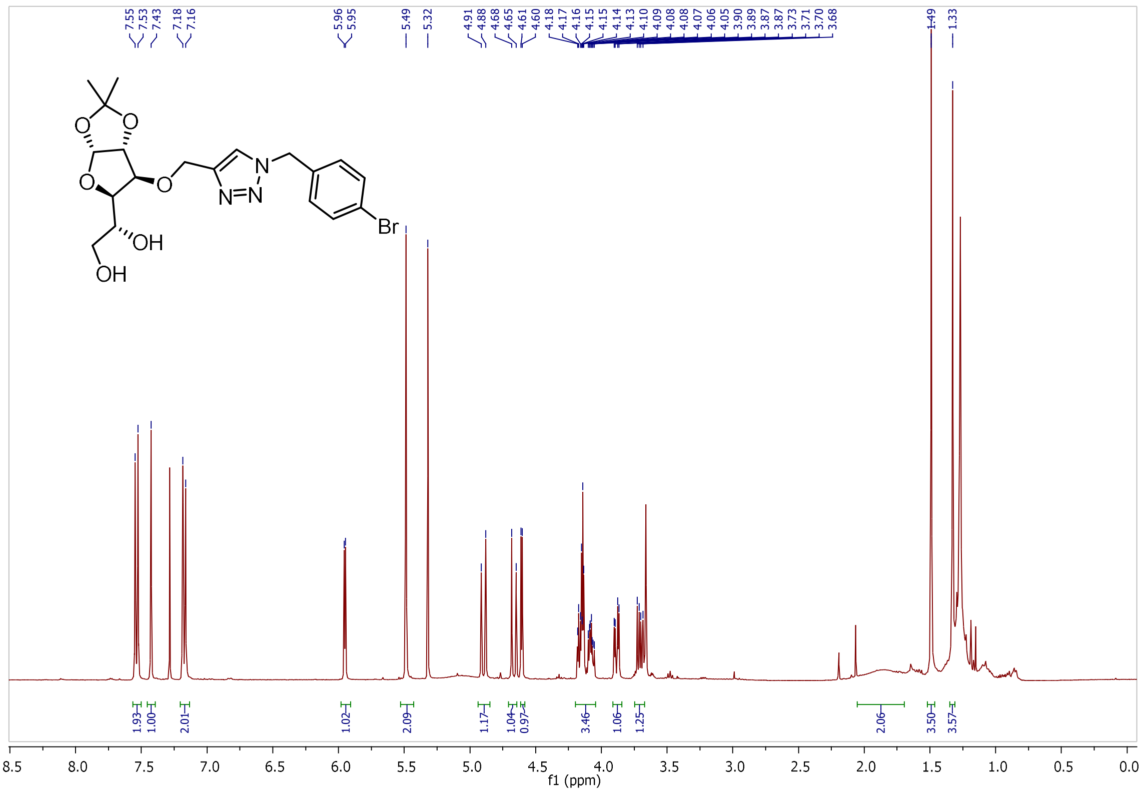

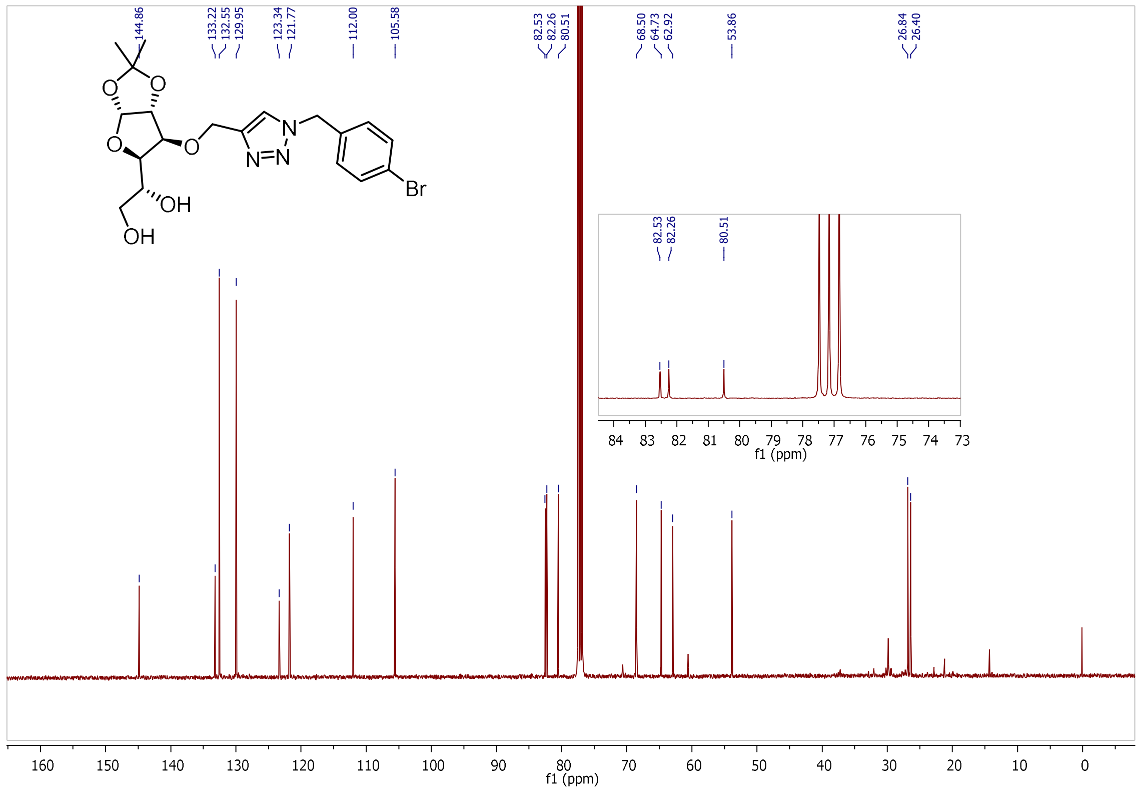

^{1}H and ^{13}C ($CDCl_3$) spectra for compounds **9d**

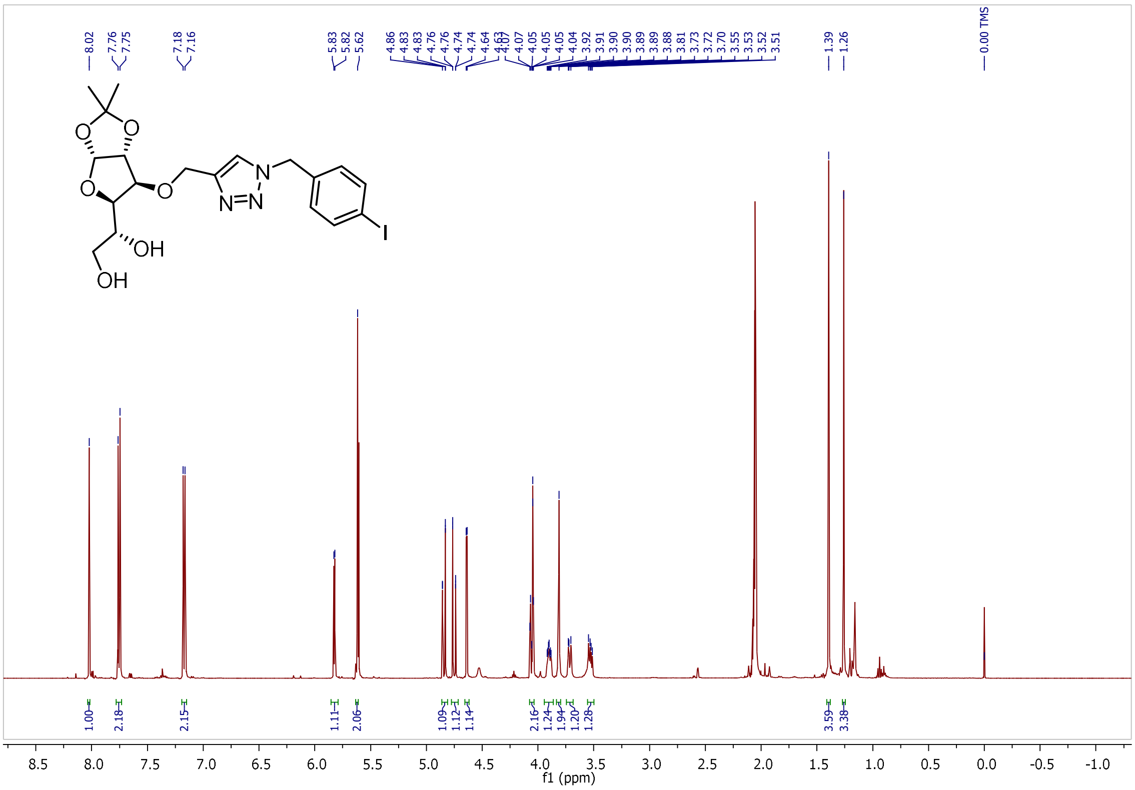

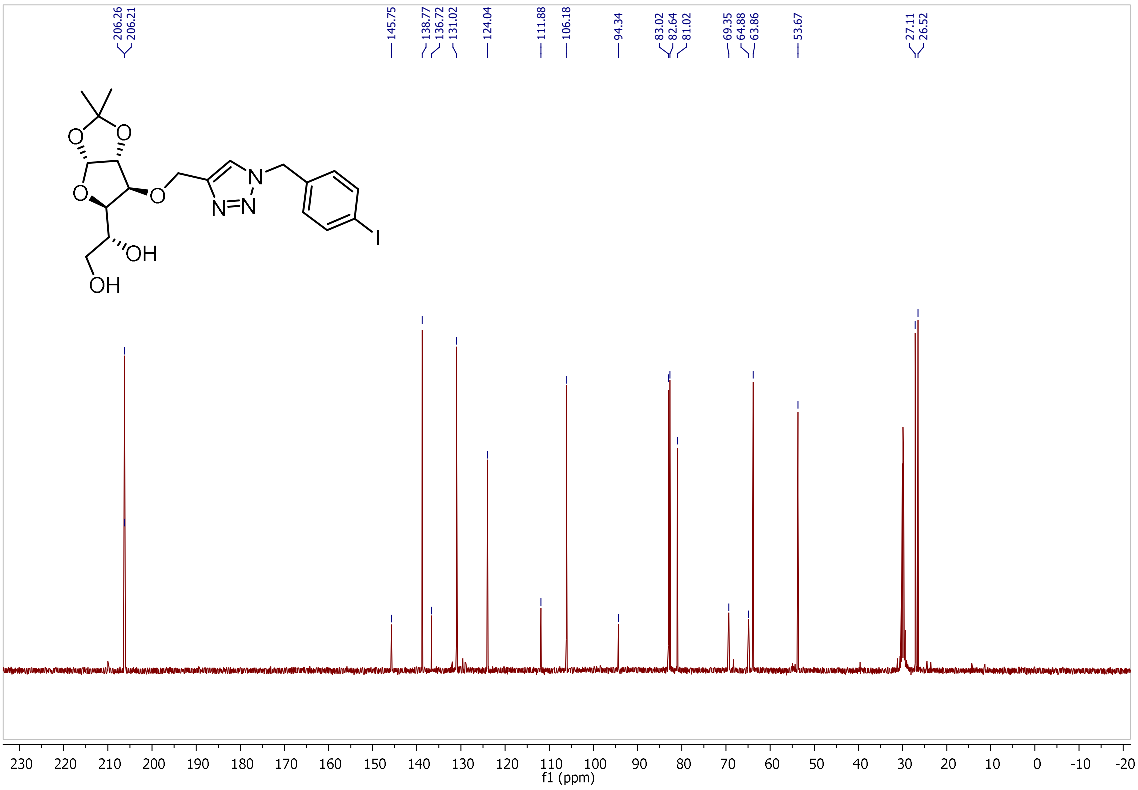

^{1}H and ^{13}C (Acetone-d_6) spectra for compounds **9e**

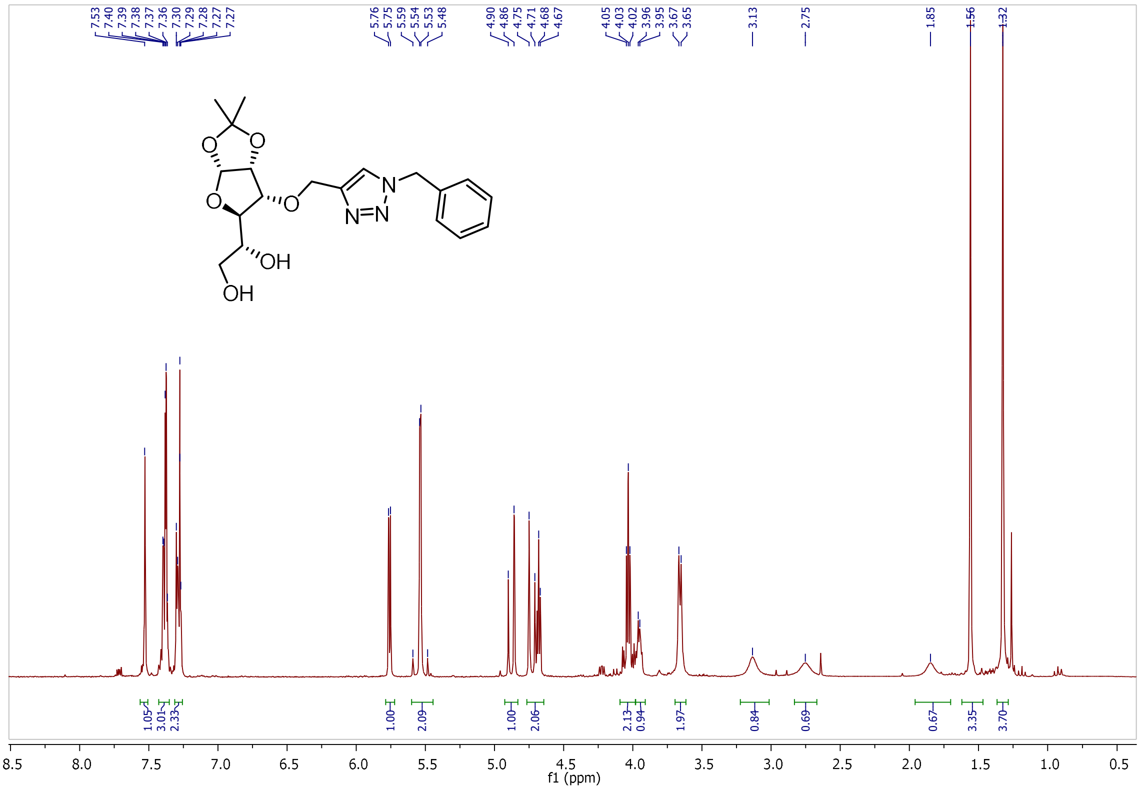

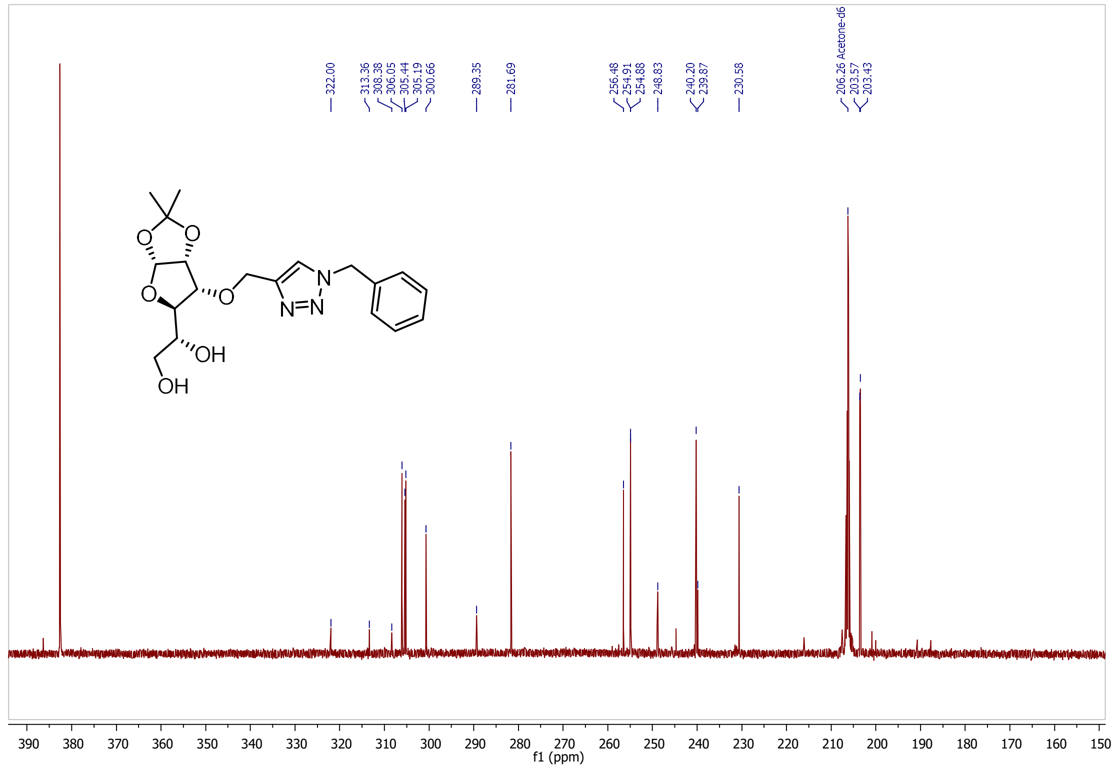

^{1}H and ^{13}C (Acetone-d_6) spectra for compounds **10a**

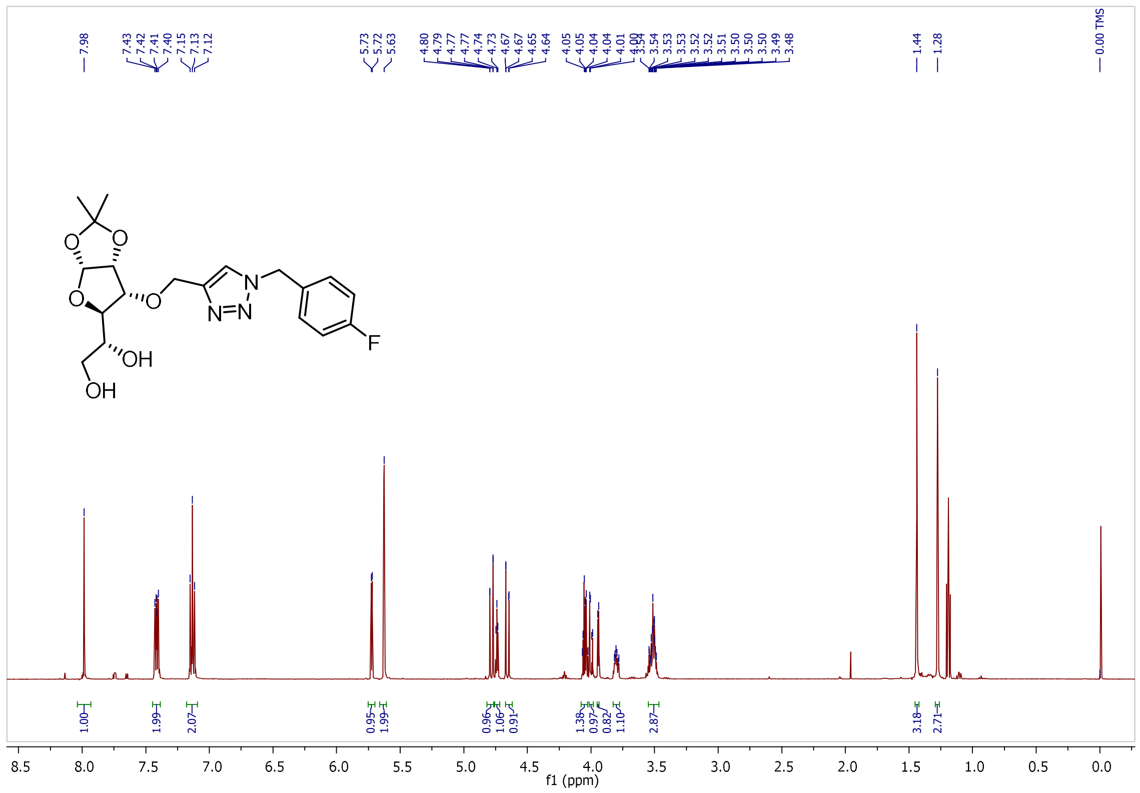

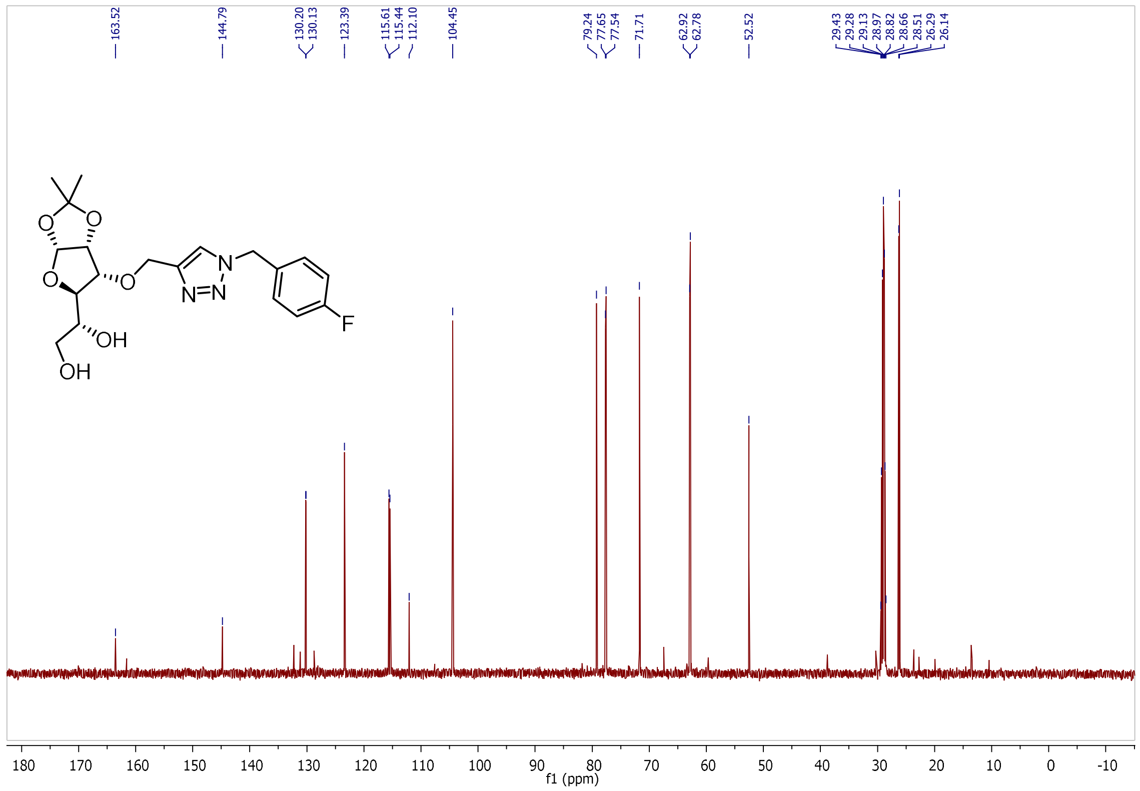

^{1}H and ^{13}C ($CDCl_3$) spectra for compounds **10b**

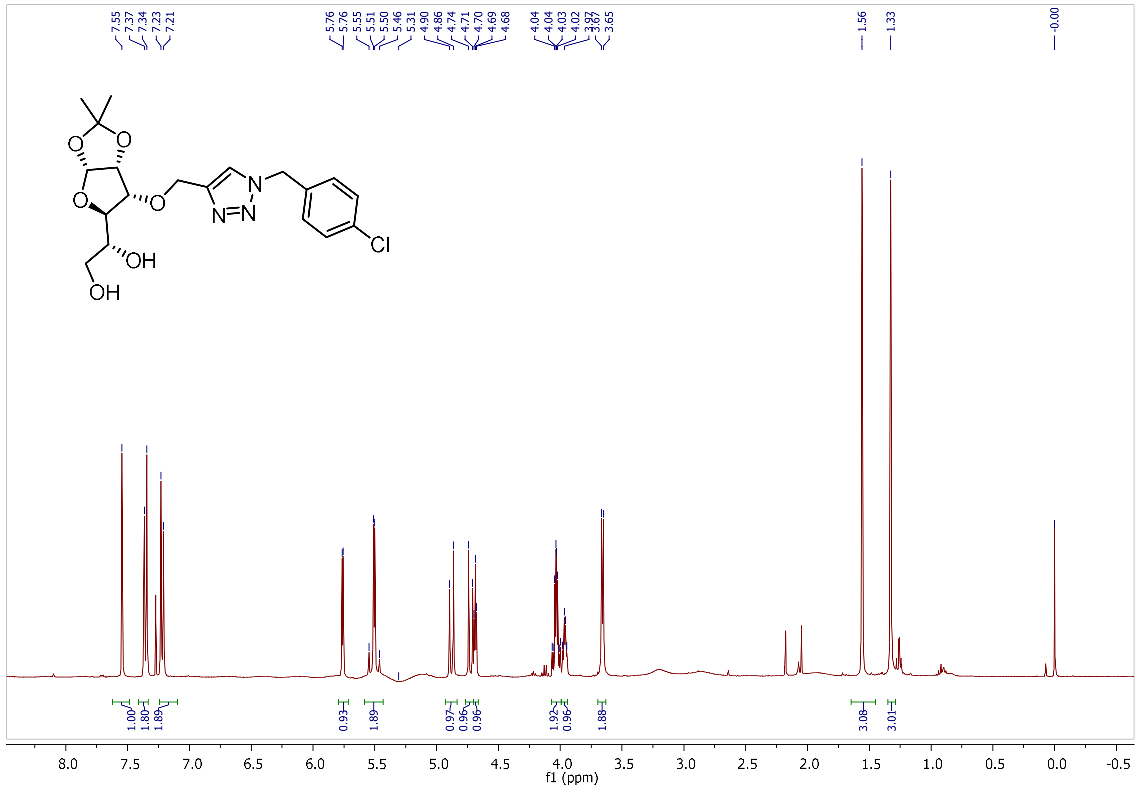

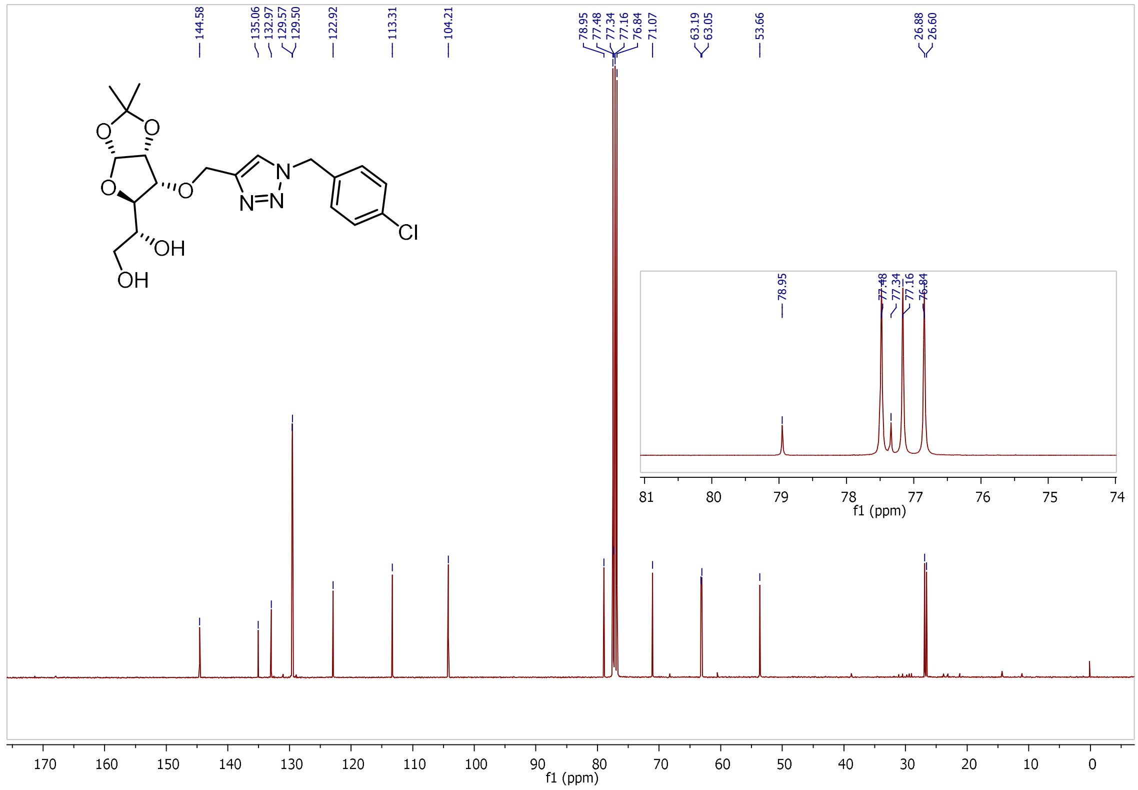

^{1}H and ^{13}C ($CDCl_3$) spectra for compounds **10c**

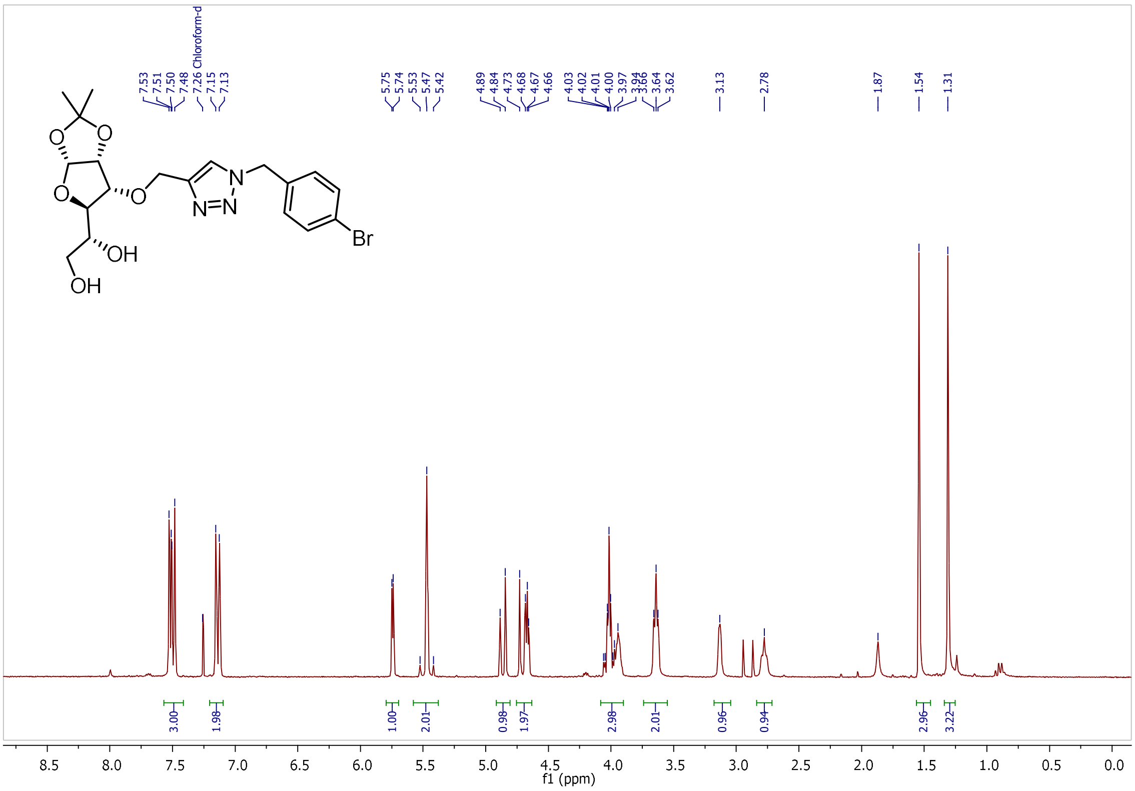

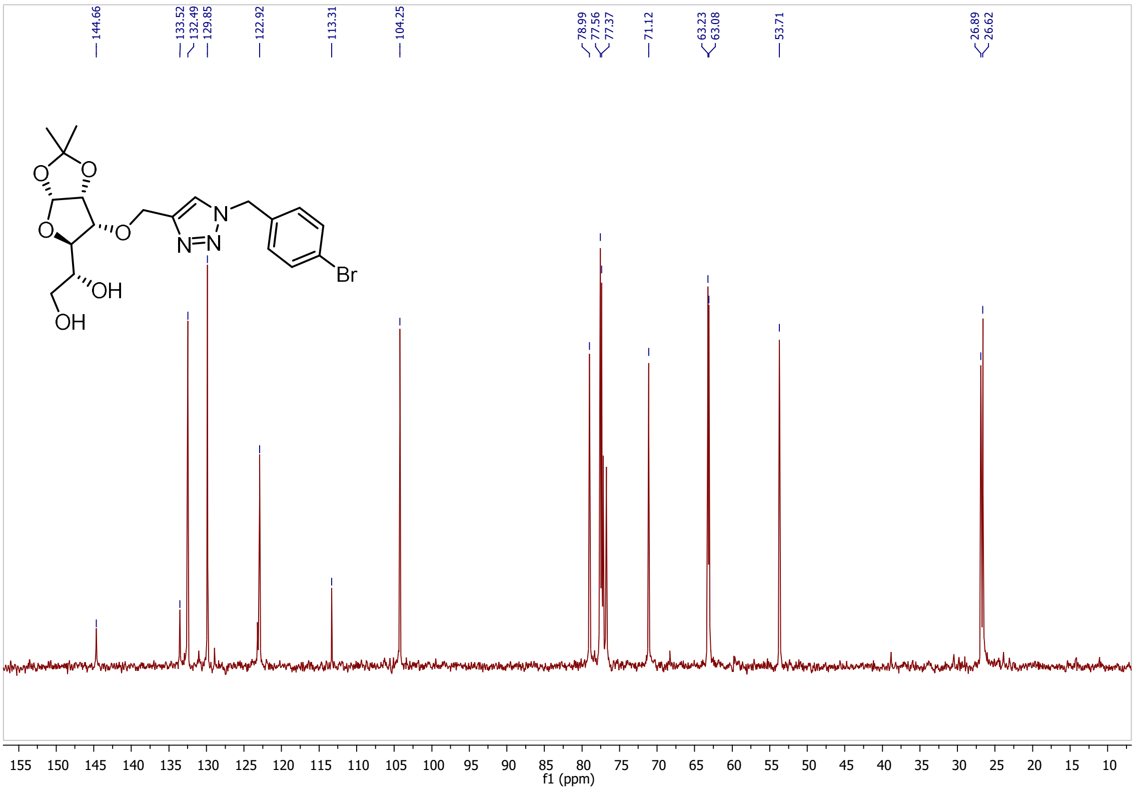

^{1}H and ^{13}C ($CDCl_3$) spectra for compounds **10d**

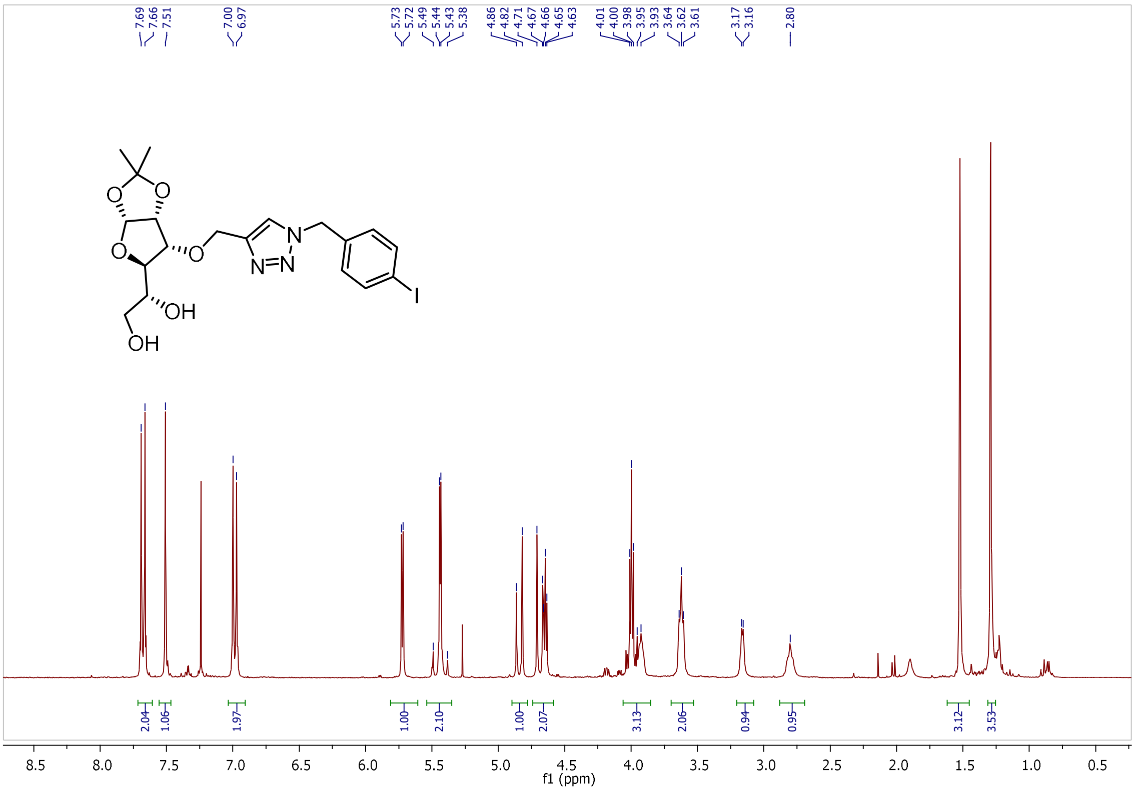

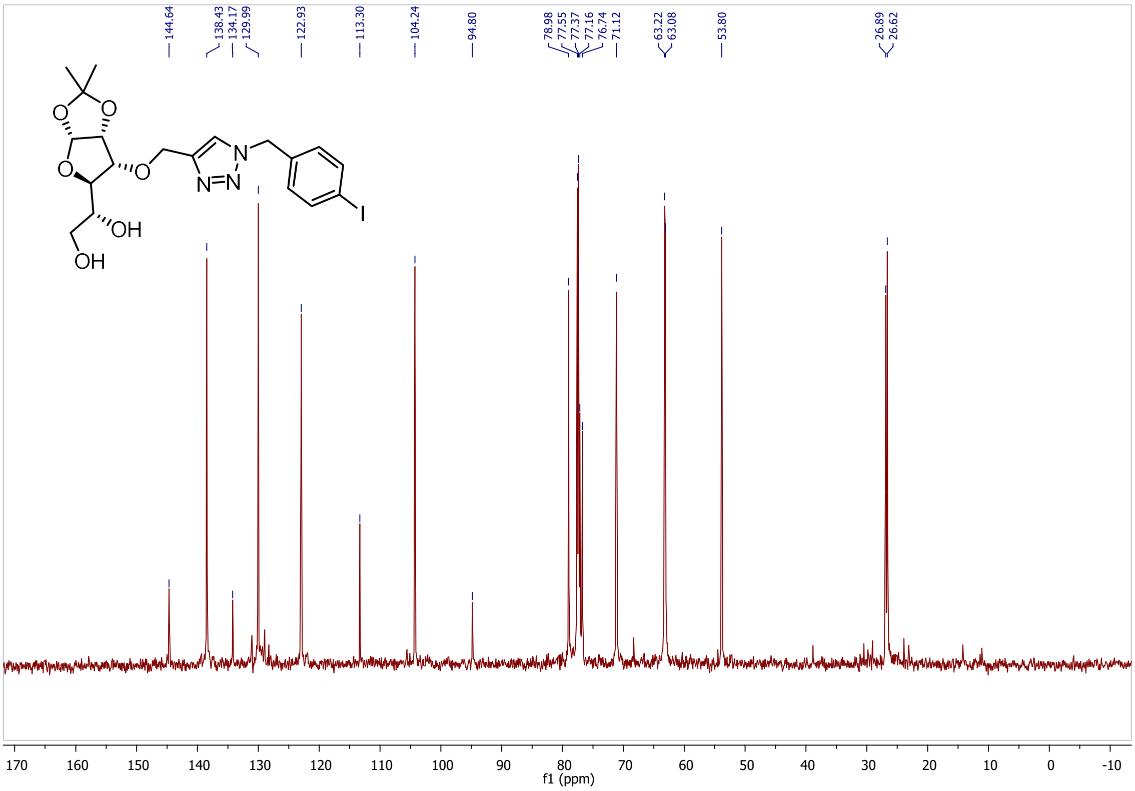

^{1}H and ^{13}C ($CDCl_3$) spectra for compounds **10e**
